# Anatomy and RNA-Seq reveal important gene pathways regulating sex differentiation in a functionally Androdioecious tree, *Tapiscia sinensis*

**DOI:** 10.1186/s12870-019-2081-7

**Published:** 2019-12-16

**Authors:** Gui-Liang Xin, Jia-Qian Liu, Jia Liu, Xiao-Long Ren, Xiao-Min Du, Wen-Zhe Liu

**Affiliations:** Key Laboratory of Resource Biology and Biotechnology in Western China (Northwest University), Ministry of Education, School of Life Science, Northwest University, Xi’an, 710069 Shaanxi China

**Keywords:** Morphology and anatomy, Androdioecious tree, *Tapiscia sinensis*, Sex differentiation, Transcriptome analysis

## Abstract

**Background:**

Gametogenesis is a key step in the production of ovules or pollen in higher plants. The sex-determination aspects of gametogenesis have been well characterized in the model plant *Arabidopsis*. However, little is known about this process in androdioecious plants. *Tapiscia sinensis* Oliv. is a functionally androdioecious tree, with both male and hermaphroditic individuals. Hermaphroditic flowers (HFs) are female-fertile flowers that can produce functional pollen and set fruits. However, compared with male flowers (MFs), the pollen viability and number of pollen grains per flower are markedly reduced in HFs. MFs are female-sterile flowers that fail to set fruit and that eventually drop.

**Results:**

Compared with HF, a notable cause of MF female sterility in *T. sinensis* is when the early gynoecium meristem is disrupted. During the early stage of HF development (stage 6), the ring meristem begins to form as a ridge around the center of the flower. At this stage, the internal fourth-whorl organ is stem-like rather than carpelloid in MF.

A total of 52,945 unigenes were identified as transcribed in MF and HF. A number of differentially expressed genes (DEGs) and metabolic pathways were detected as involved in the development of the gynoecium, especially the ovule, carpel and style. At the early gynoecium development stage, DEGs were shown to function in the metabolic pathways regulating ethylene biosynthesis and signal transduction (upstream regulator), auxin, cytokinin transport and signalling, and sex determination (or flower meristem identity).

**Conclusions:**

Pathways for the female sterility model were initially proposed to shed light on the molecular mechanisms of gynoecium development at early stages in *T. sinensis.*

## Background

Angiosperm plants exhibit a wide variety of breeding systems [1, 2]. Among them, hermaphroditism is thought to be the ancestral breeding system in angiosperms [3, 4], and dioecy appears to have evolved from hermaphroditism multiple times [1, 5–7]. Although two main pathways for the evolution of dioecy have been proposed, dioecy that evolves from an ancestral hermaphrodite or from a monoecious species is considered to be the most common [1, 6–9]. On this pathway, it is believed that hermaphroditic individuals coexist with unisexual mutants that have lost either their male (gynodioecy) or their female (androdioecy) function [8]. Among the variety of reproductive mechanisms in flowering plants, one permutation – androdioecy (mixtures of males and hermaphrodites) – is distinguished by its rarity and represents less than 0.005% of all angiosperms [10]. However, androdioecy is thought to be relatively unstable; models of mating system evolution predict that androdioecy should be a brief stage between hermaphroditism and dioecy (separate males and females), or vice versa [5, 8, 9, 11–15]. Loss of function is not a big challenge. The biggest challenge to female sterilty is due to natural selection. Typically if an individual looses female fertility, its fitness will be halved, and natural selection should act against it, eliminating the mutation.

Separate sexes have evolved on numerous independent occasions from hermaphroditic ancestors in flowering plants [16]. Mechanisms controlling sex can be genetic, epigenetic (physiological and environmental) [17–24], or plant hormones [2, 25]: (1) Genetic control of sex determination is driven by chromosomes and sex determining genes in the dioecious plants. According to Heikrujam et al. (2015), genetic sex determination may be due to a single locus or multiple loci either unlinked or tightly linked on autosomes [2]. For example, in persimmon, only a single locus is responsible for sex determination [26, 27] while the evolution of separate sexes in asparagus invokes sterility mutations at two linked loci [27, 28]. (2) Hormonal regulation of unisexual flower development [29]. Unisexual flower development in *Cucumis sativus* (cucumber) and *Cucumis melo* (melon) is regulated by the interaction of environmental cues, plant hormones and genetic factors that differentiate gender phenotypes [24, 25, 30]. In kiwifruit, a Y-encoded suppressor of feminization, *Shy Girl*, arose via lineage-specific duplication of a cytokinin response regulator [29]. At present, in some plant species, such as *Arabidopsis* [31, 32], cotton [33], rice [34], tomato [35], *Silene latifolia* [36, 37], *Zea mays* [38–40], *Diospyros lotus* [26], and cucumber [25], significant progress in elucidating the molecular mechanism of pistil development and sex determination has been achieved [24–26]. Molecular studies in these species have led to important advances in our understanding of plant sex determination and differentiation [25, 26, 41]. For example, *ANT* promotes ovule primordium growth; the *CUP*-*SHAPED COTYLEDON* genes (CUCs) play a role in the establishment of the ovule primordium boundaries; when the functions of *stk*, *shp1*, and *shp2* were lost in a triple mutant, fewer ovules were initiated, and ovule development was severely disrupted. Even more remarkably, the ABCDE model of flower determination and development has indicated that a specific class of MADS-box genes are key regulators of pistil development [42], such as *AGAMOUS* (*AG*), *APETALA 2* (*AP2*), *BELL 1*, *INNER NO OUTER* (*INO*), *AINTEGUMENTA* (*ANT*), *SPOROCYTELESS* (*SPL*/*NZZ*), and *SUPERMAN* (*SUP*) [43, 44].

**Fig. 1:**
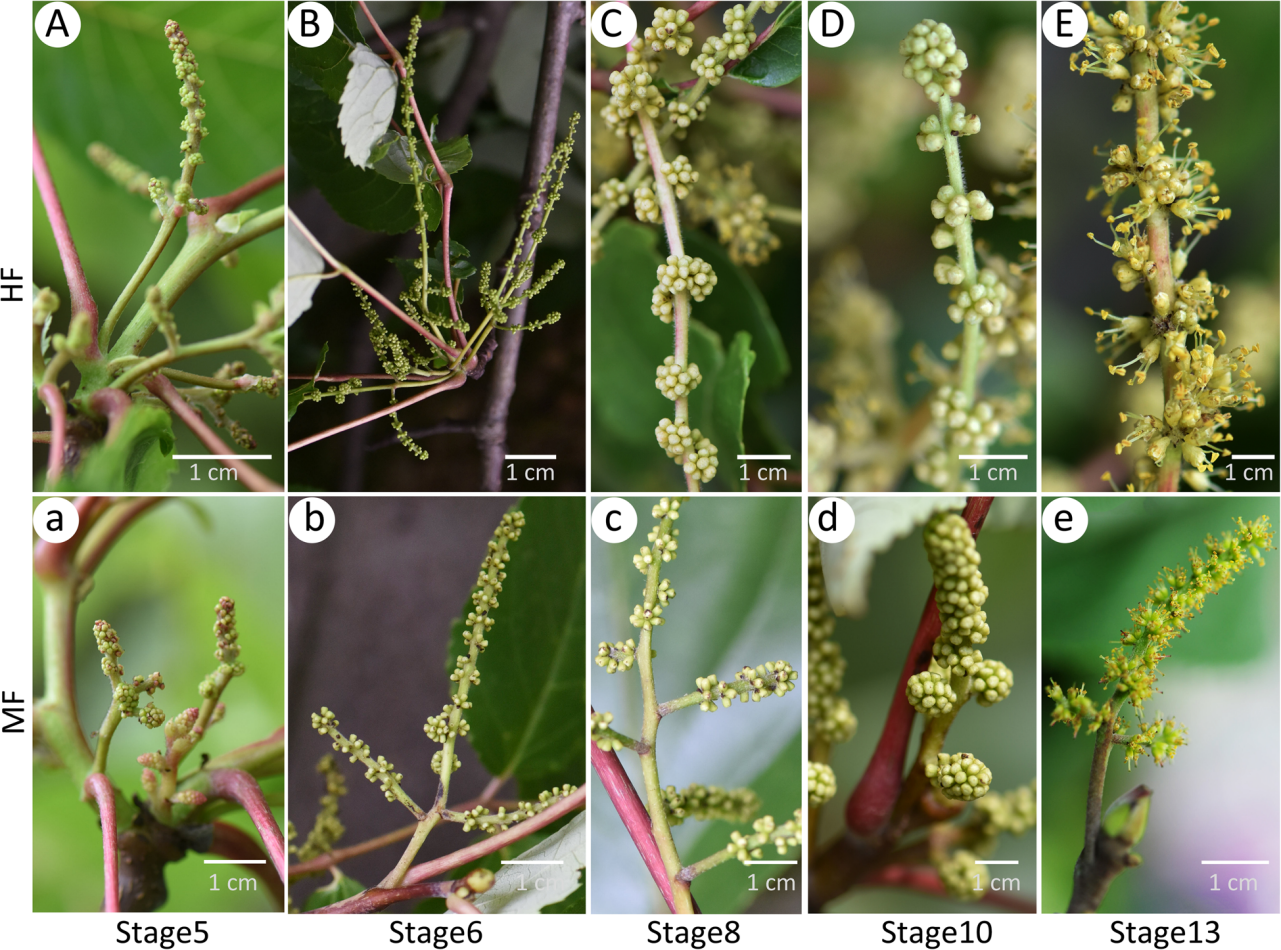
Photographs showing the developmental stages of the male and bisexual flowers. (**a**–**e**) Male inflorescences at stages 5, stage 6, stage 8, stage 10 and stage 13, respectively. (**a**–**e**) Bisexual inflorescences at stage 5, stage 6, stage 8, stage 10, and stage 13, respectively. Bar=1 cm

**Fig. 2:**
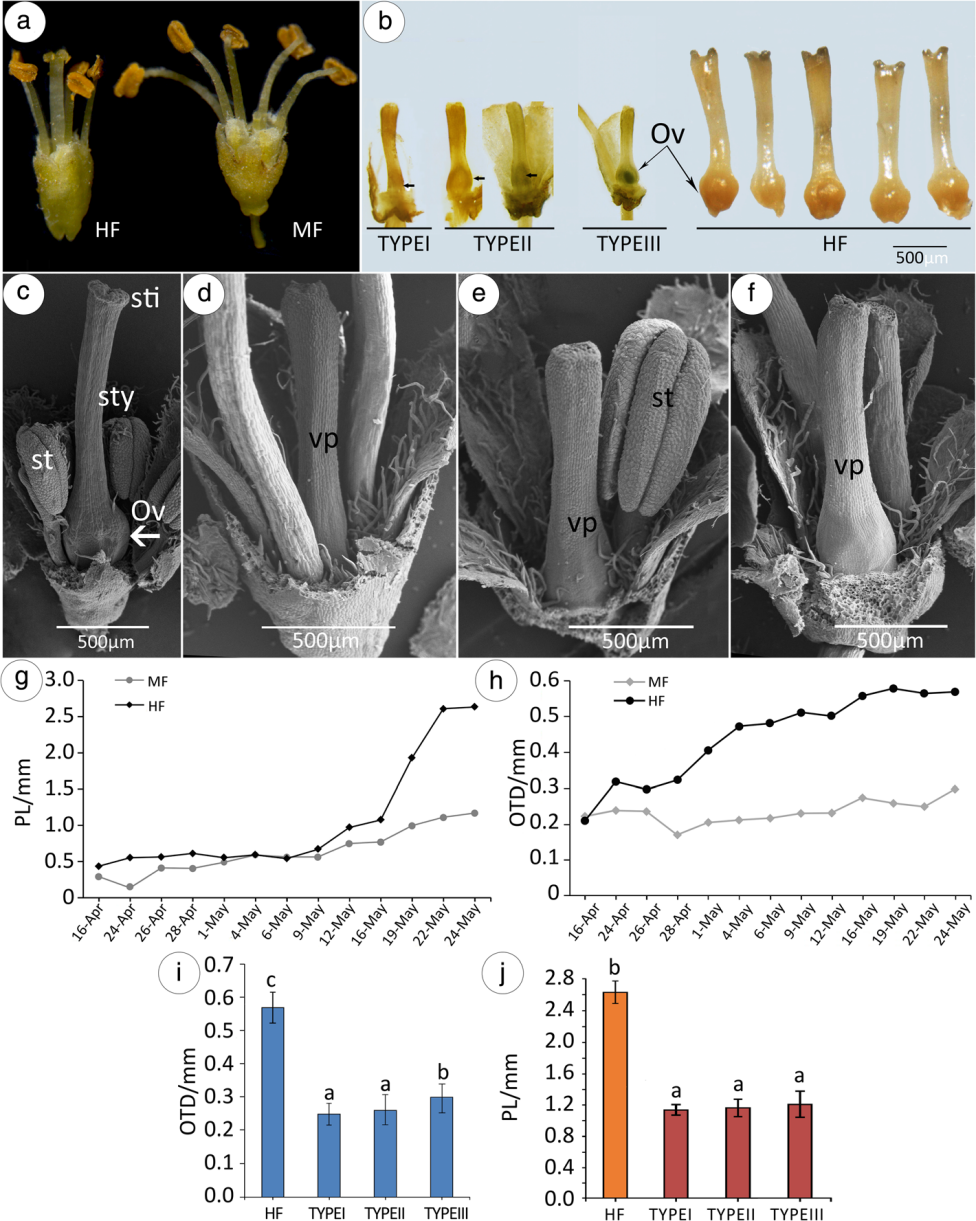
Characteristics of the flowers. **a** Morphology of HF (left) and MF (right). **b** Compared with HF (right), there are three types of gynoecia in MF. **c** Scanning electron microscope (SEM) observation of HF. **d**-**f** SEM observation of three types of MF. **g** Change in pistil length (PL) over time. **h** Change in ovary transverse diameter (OTD) over time; **i** OTD in HF - Type I, Type II, and Type III comparison. **j** PL in HF - Type I, Type II, and Type III comparison. Each data point represents the mean value of 30 technical replicates

Recent advances in the evolution of androdioecy showed that androdioecy arose from hermaphroditism because some male flowers have non-functional pistils, and that these residual organs are common in androdioecious and gynodioecious species that arose from hermaphroditism [13, 45–49], and that in species where androdioecy arose from dioecy, non-functional pistils are absent [50, 51]. In the functionally androdioecious *Tapiscia sinensis* Oliv. (Tapisciaceae) [52–54]*,* the male individuals have pistillode, some of which have a shorter column and abortive ovule, while the vast majority of pistils lack ovaries (Figs. 1 and 2a). Thus, this plant might provide a perfect model of male individuals that originated from an ancestral hermaphrodite. Furthermore, the stage at which pistil abortion occurs and sex differentiation is determined by sex chromosomes, sex-determining genes, or hormone regulation remians unclear.

To determine the molecular mechanism of female sterility in male individuals, we compared the developmental anatomy and performed transcript profiling using the RNA-Seq technique of the gynoecia of the HF and MF. A number of candidate genes and related pathways were revealed, which provided new insights into the genetic and biochemical controls for early stages of pistil development, and our results will be helpful in shedding light on the mechanism of sex differentiation in the functionally androdioecious trees of *Tapiscia*.

## Results

### Floral morphology

**Fig. 3:**
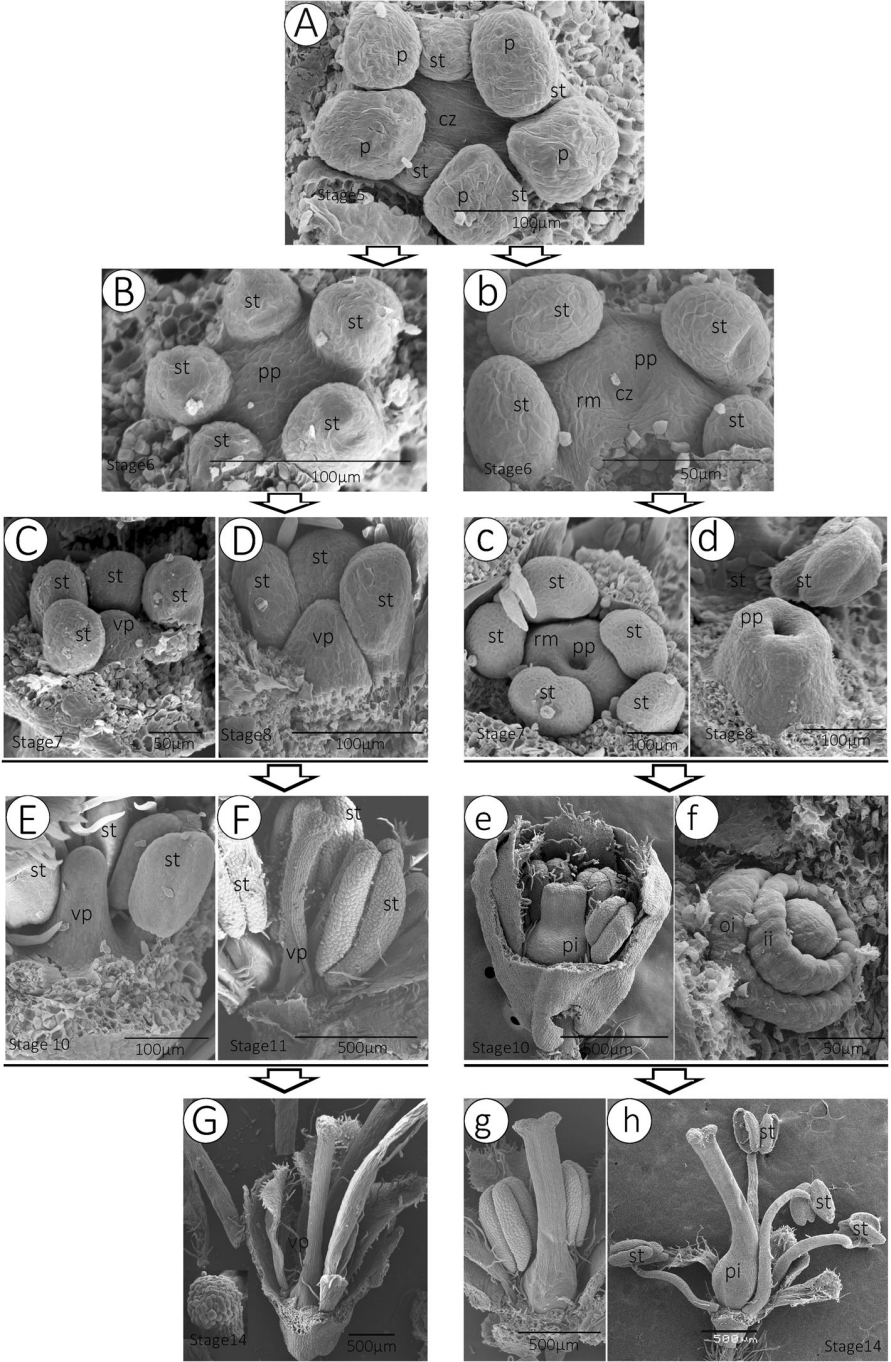
Comparison of the sex differentiation between Hermaphrodite Flowers and Male Flowers. In *T. sinensis*, male and bisexual flowers have the same calyx initiation, petal initiation and stamen initiation pattern (from stage 1 to 5), so we begin to show their differences from stage 5. **a** The morphology of 5 petals and 5 stamens. **a**-**b**~**g** The morphology of HF: **a**, **b**, **c** Two or three gynoecium primordia begin to form as a ridge (the ring meristem) around the center of the flower; **c**, **d** at stage 7, the gynoecium grows as a hollow tube; at stage 8, the gynoecium tube grows taller and wider; during stage 10, ovule primordiu were observable; **g**, **h** at flowering time, the gynoecium becomes ready for fertilization, and while the stamens extend out of the petals, fertilization occurs. **a**-**b**~**g** The morphology of MF: **a**, **b** at stage 6, the gynoecium primordium also begins to form, but the central zone or gynoecium primordium on the flower apex was fused as a small bulge; **c**~**e** Abnormal pistil primordium grows rapidly, and finger-like. **f**, **g** The pistil was characterized by a solid, stem-like structure. vp, vestigial pistil; oi, outer integument; ii, inner integument; rm., ring meristem; pp., pistil primordium; st, stamen; pi, pistil; cz, central zone

The morphology of MFs is funnel-shaped, whereas that of HFs is water goblet-shaped (Fig. 2a) and have a normal pistil with a stigma, a style, and an ovary (Fig. 2c). In comparison with HF, the gynoecia of MF were slow-growing and much shorter from the early development stage to flowering (Fig. 2b, g). In natural populations, the MFs have three types (Fig. 2b, d-f). In comparison with Type III, the ovary transverse diameters (OTD) in Type I and Type II is smaller (*p* < 0.05) (Fig. 2i), while no significant differences exist among their pistil lengths (PL) (Fig. 2j). Correspondingly, the flowering duration of MF plants was prolonged until complete fertilization of HF because they had no seed setting and produced more flowers. MF had shorter pistils, and smaller ovules than HF. At flowering time, MF bloomed at a PL of 1.17 ± 0.17 mm (Fig. 2g) and an OTD of 0.518 ± 0.05 mm (Fig. 2h). In contrast, HF bloomed at a PL of 2.63 ± 0.54 mm (Fig. 2j) and an OTD of 1.15 ± 0.08 mm (Fig. 2i). The OTD (T = -7.102, df = 12, *P* < 0.01) and the PL (T = − 2.876, df = 12, *P* < 0.05) were clearly shorter in MF than in HF. Remarkably, in MFs, the PL and OTD remain unchanged when the bud vertical diameter grew to 0.75 ± 0.05 mm (stage 9, May 16th) (Fig. 2d). Later, some limited growth may continue from stage 9 to flowering for the carpel in MFs, which is often only one-quarter to one-half the full length that in the HFs (Fig. 3j).

The SEM results showed that there were no obvious differences observed between MF and HF before stage 5 (Fig. 3a). However, at stage 6, in HFs, the gynoecium primordial begins to form as a ridge of raised cells around the center of the flower apex (Fig. 3a, b, c). At stage 7, the gynoecium grows as a hollow tube (Fig. 3c, d); At stage 8, the gynoecium tube grows taller and wider, the gynoecium continues to grow upward to form a continuous hollow cylinder, and placenta primordia were observable; during stage 10, in the centre of the receptacle, ovule primordium were observable (Fig. 3e, f); at the flowering time (stages 11–14), the gynoecium becomes ready for fertilization, and when the stamens extend out of the petal, fertilization occurs (Fig. 3g, h).

In most MFs, during stage 6, the gynoecium primordium began to form (Fig. 3b), but the central zone was fused as a hemispherical bulge and no differentiation of the ring meristem is present (Fig. 3c, d), which was obviously different from the ring meristem in HFs (Fig. 3b, c). The pistil structure of the MF was characterized by the formation of a solid, stem-like structure (Fig. 3e-g). The key stage at which ovule abortion occurred was stage 6 (Fig. 3b, B).

### Structure of the flower

**Fig. 4:**
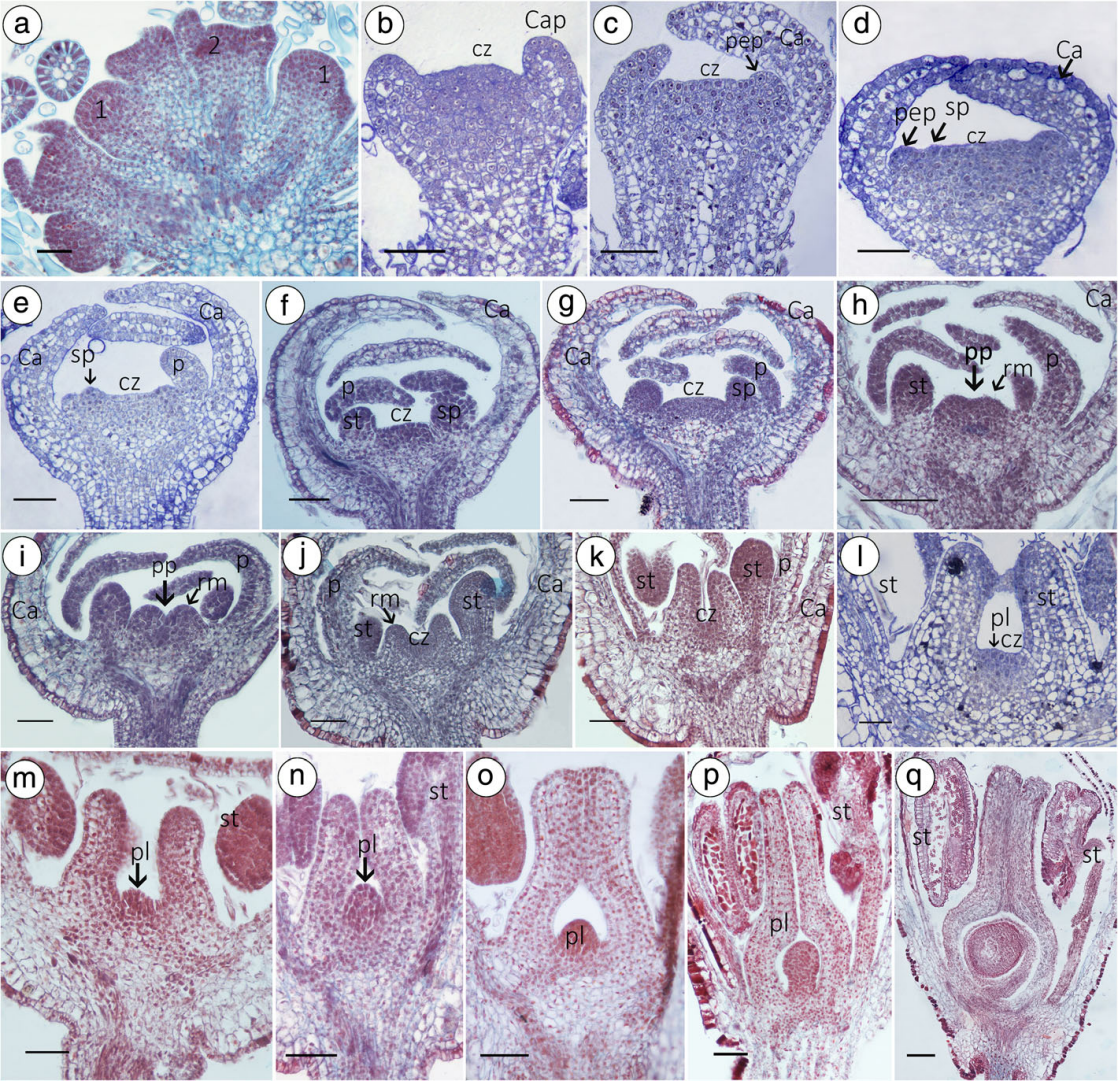
Developmental anatomy of the HF. **a** the flower primordium initiated. **b** The calyx primordium initiated. **c** When the calyx covered the central zone of the flower, the petal primordium initiated. **d**, **e** The cetral zone of the flower becomes broad, then the stamen primordium intiated. **f**, **g** The central zone (cz) of the flower apex raised as a platform. **h**, **i** During stage 6, the sepals enclose the central zone of the flower bud and the carpel primordia begin to form, where the raised cells surround the floral apex as a ridge. **j**, **k** Gynoecium continues to grow upward to form a continuous hollow cylinder. **l** The central zone becomes broad, where the placenta primordia will be formed; **m**, **n** At stage 7, the medial stamens become stalked at their bases, the gynoecium grows as a hollow tube, and placenta primordia are observable. **o** Rapid growth of the placental primordia and style, and the unilocular formed. **p** Period of the formation of ovule primordium. **q** In the centre of the ridge, ovules will form. Bar = 100 μm. Cap, calyx primordium; pep, petal primordium; cz, central zone; sp., stamen primordium; rm., ring meristem; st, stamen; p, petal; pep, petal primordium; pl, placenta primordium

In *T. sinensis*, the course of individual flower development can be divided into five phases: early differentiation, calyx initiation, petal initiation, stamen initiation, and carpel initiation. The perianth was the first organ to be formed on the floral apex (Fig. 4a, b). Sepals differentiated concurrently and curved to cover the central zone (Figs. 3a and 4c). In late stage 5, petal and stamen primordia are initiated (Fig. 4d). As the stamens formed, there was a bud vertical diameter of 76.92 ± 1.98 μm, and the floral apex became broadly concave (Fig. 4e). The central zone (floral meristem) of the flower bulges out to form a platform on which the gynoecium primordia will develop (Figs. 4f, g and 3b). At stage 6, the sepals enclose the flower bud, and two or three carpel primordia begin to form, with raised cells around the floral apex constituting a ridge (ring meristem) (Fig. 4h, i). At stage 7, the medial stamens become stalked at their bases, and the ring meristem grows as a hollow tube (vertical section) (Fig. 4j~l); at stage 8, the placenta primordium is observable (Fig. 4m~o); then, the ovule forms from the placenta (Fig. 4p, q). The two or three carpels gradually fused with each other in hermaphrodites and eventually developed into a normal pistil with a stigma, a style, and an ovary (Additional file 4: Figure S1).

**Fig. 5:**
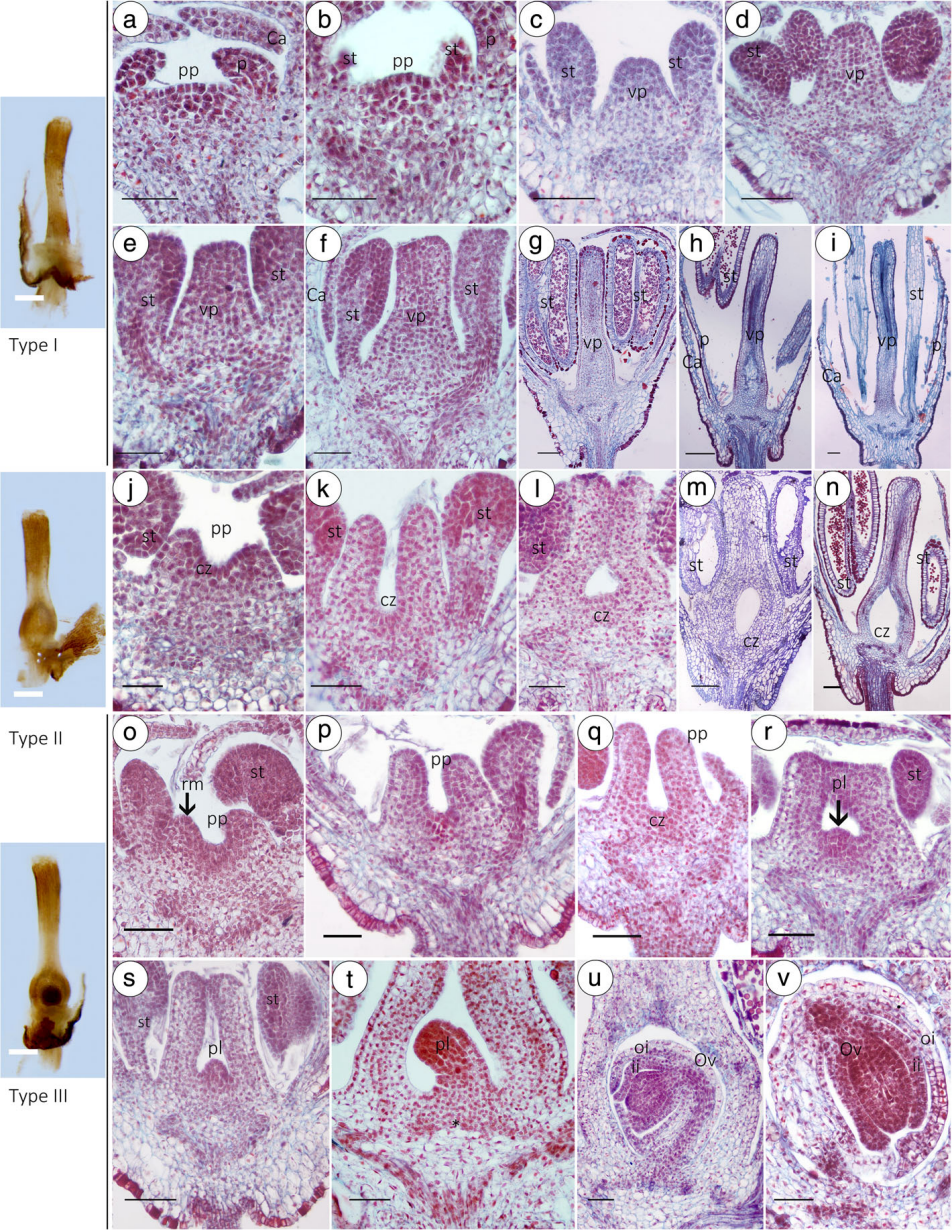
Developmental anatomy of the MF. Three types of gynoecia in MF: Type I, Gynoecia without ovary (**a**-**i**); Type II, ovules were arrested at stage 6 (**j**-**n**); Type III, the MF have a well-developed pistil that is sterile (**o**-**v**), particularly. Bar=100 μm

From stage 1 to stage 5, hermaphroditic individuals and males have the same developmental process and morphological structures (Fig. 4a-k). However, after stage 5, the carpel primordium of the centre of the floral apex in MFs exhibits different patterns from that of HFs. Longitudinal sections of MF from the same tree showed that there are three types of pistils. Type I: Most of the MFs without ovules or ovaries were characterized by the formation of a solid, stem-like residual pistil (Fig. 5a-i). Type II: A tiny minority (1.6%) of MF had one ovary but no ovule structure (Fig. 5j-n). Type III: MFs have a well-developed pistil that is sterile (3.33%); the ovary has one chamber that has one ovule that has double integuments and is crassinucellate and anatropous (Fig. 5o-u); however, when the MF falls off after maturation, termination of embryo sac development occurs at the triad stage (Fig. 5v; Additional file 4: Figure S1).

**Fig. 6:**
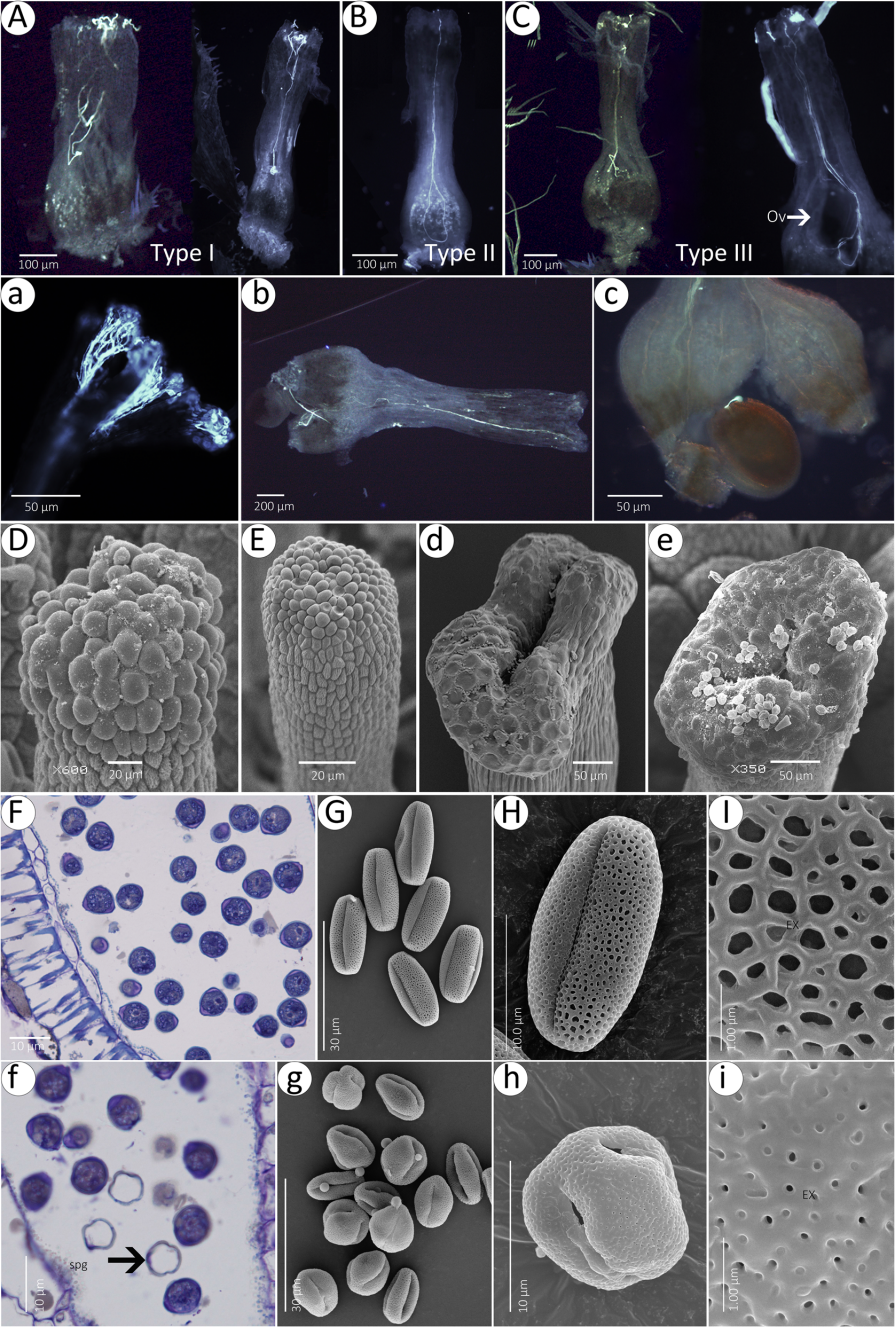
Pollen germination, pollen tube elongation and papilla cells in MF (**a**-**e**) and HFs (**a**-**e**). **a**-**c** The pollen tubes are screw-shaped and penetrate into papilla cells in the three types of MF at 24 h after self-pollination. **a**-**c** At 24 h after self-pollination, the pollen tubes passed into the micropyle of the ovule of HF. **d**, **e** Papilla cells of undivided stigma. **d**, Bilobed stigma. **e**, Trilobate stigma. **f**, Anther and pollen grain sections of HF; sterile pollen grains (spg) were observed. **g**, Globose pollen grains in HF. **h**, Globose pollen grain; **i**, Detail of perforate tectum. **f**, Anther and pollen grain sections. **g**, Olivary pollen grains. **h**, Olivary pollen grain in MF; **i**, Detail of reticulate tectum

A pollination analysis found that a few pollen grains from self or HF could germinate on MF stigmas 6 h after pollination. Remarkably, compared with HF (Fig. 6a-c), in MF, although pollen tubes could grow in a spiral manner over the surface of the papilla cells, some of them penetrated into stigmas or styles and then succeeded in reaching the abnormal ovules 24 h after pollination (Fig. 6a-c). Further observation showed that the papilla cells on MF stigmas (Fig. 6d, e) were similar to HFs at the maturation stage (Fig. 6d, e). The anther and pollen grain sections show that many sterile pollen grains (spg) were observed in HF (Fig. 6f) but not in MF (Fig. 6f). There are two types of pollen in hermaphroditic flowers (Fig. 6g), while there is only one type of pollen in MF (Fig. 6g). In contrast to the perforate tectum of the olivary pollen grains in MF (Fig. 6h, i), the pollen grains in HF have a reticulate tectum (Fig. 6h, i).

### Illumina sequencing and sequence assembly

In this project, the Illumina HiSeq™ 4000 sequencing platform was used for transcriptome sequencing, and 962,641,935 clean reads were obtained, with a mean length of 150 base pairs (bp). The average GC percentage was 44.69%. The clean reads were de novo assembled using Trinity [55] into 63,573 contigs, with a mean length of 1132 bp and an N50 of 1892 bp. Then contigs were used for sequence clustering with the software TGICL (TIGR Gene Indices clustering tools) to form unigenes [56]. The total number of unigenes was 52,945, with a mean length of 973 bp and N50 of 1768 bp. The average depth of sequence coverage was 82.42%. In all, 36,353 unigenes within 201–1000 bp, which was 57.59% percent. Additionally, 7766 unigenes (14.67%) ranged from 1001 to 2000 bp in length, while 7408 of the unigenes (13.99%) were over 2000 bp in length.

### Differentially expressed genes (DEGs) and enrichment analysis

Using the series test of cluster (STC) [57], a total of 9989 and 7326 unigenes were identified and transcribed in the M1-M2-M3 term (TERM1)_vs_H1-H2-H3 term (TERM2), respectively (Additional file 4: Figure S3). M1, M2 and M3 represent MF at stages 5, 6 and 10, respectively, while H1, H2 and H3 represent bisexual flowers at stages 5, 6 and 10, respectively. In TERM1, the expression patterns of 13,761 genes were analysed, and eight model profiles were used to summarize (Additional file 4: Figure S2); five expression patterns of genes showed significant *p*-values (*p* < 0.05) marked with coloured boxes, including profile 3, profile 4, profile 5, profile 6, and profile 7 (Additional file 4: Figure S3). In TERM2, the expression patterns of 16,130 genes were analysed, and eight model profiles were used to summarize (Additional file 4: Figure S2); three expression patterns of genes showed significant *p*-values (*p* < 0.05) marked with coloured boxes, including profile 1, profile 6, and profile 7 (Additional file 4: Figure S3). Each box represents a model expression profile with the model profile number and p-value. To further clarify the functional differences between MF and HF at stage 6, using a false discovery rate (FDR) ≤ 0.05 and the absolute value of |log2 ratio| ≥ 1 as criteria, 9623, 14,347 and 12,049 unigenes exhibited significantly different expression levels in M1_vs_ H1, M2 _vs_H2, and M3_vs_H3, respectively (Additional file 4: Figure S4A-C).

To determine whether the DEGs between MF and HF are significantly related to specific pathways or biological function, GO and KEGG databases were used for enrichment analysis (Additional file 4: Figure S4D-F). For TERM1, TERM2, M1_vs_H1 DEGs, the GO terms that showed enrichment included “flavonoid biosynthetic process” (GO:0009813), “secondary metabolic process” (GO:0019748), “hormone biosynthetic process” (GO:0042446), “cytokinin biosynthetic process” (GO:0009691), “glycoside biosynthetic process” (GO:0016138), and “regulation of fertilization” (GO:0080154) (Additional file 3). In contrast, for TERM1, TERM2, M2_vs_H2, the enriched classification terms were “cell wall organization or biogenesis” (GO:0071554), “external encapsulating structure organization” (GO:0045229), “glucose metabolic process” (GO:0006006), “embryo development” (GO:0009790), “response to inorganic substance” (GO:0010035), and “response to chemical” (GO:0042221) (Additional file 3).

**Fig. 7:**
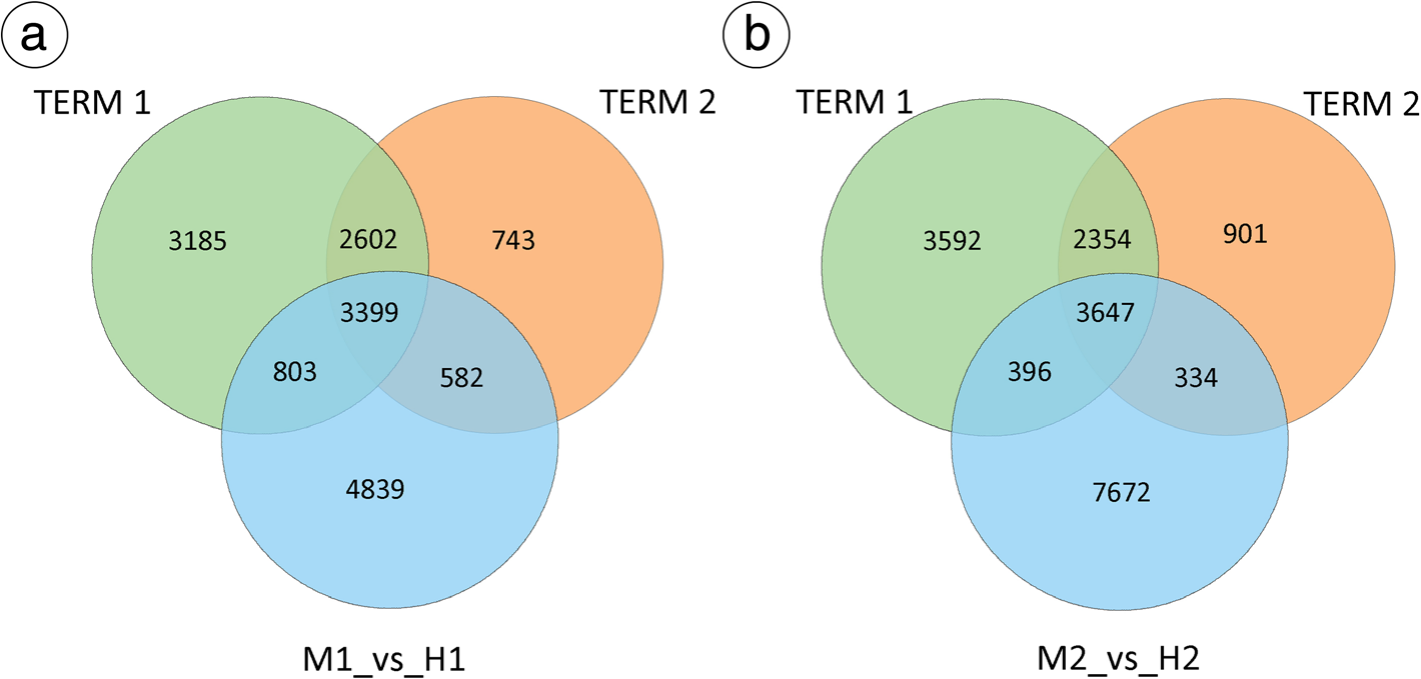
Unique and shared DEGs among different stages for both HF and MF. **a** The number of DEGs among TERM1, M1_vs_H1, TERM2. **b** The number of DEGs among TERM1, M2_vs_H2, TERM2. Venn diagrams were drawn using a online tool Venn Diagrams (http://bioinformatics.psb.ugent.be/webtools/Venn/)

Pathway assignment was carried out by KEGG to analyse the biological functions of DEGs. For the TERM1, TERM2, and M1_vs_H1 comparison (Fig. 7a), pathways related to “Phenylpropanoid biosynthesis” (ko00940), “Plant-pathogen interaction” (ko04626), “Plant hormone signal transduction” (ko04075), “Glucosinolate biosynthesis” (ko00966), “Diterpenoid biosynthesis” (ko00904), “Starch and sucrose metabolism” (ko00500), “Zeatin biosynthesis” (ko00908), “Ribosome” (ko03010), and “Flavonoid biosynthesis” (ko00941) were enriched (Additional file 3). For the TERM1, TERM2, and M1_vs_H1 comparison (Fig. 7b), the main pathways included “Glycolysis/Gluconeogenesis” (ko00010), “Pentose and glucuronate interconversions” (ko00040), and “Tryptophan metabolism” (ko00380), “Citrate cycle (TCA cycle)” (ko00020), “Starch and sucrose metabolism” (ko00500), “Plant hormone signal transduction” (ko04075), and “Zeatin biosynthesis” (ko00908) (Additional file 3). The enrichment analysis revealed a total of 63 DEGs related to plant hormone signal transduction. Thus, the process of “plant hormone signal transduction” was concluded to be a putative pathway affecting *T. sinensis* flower development (Additional file 3).

In brief, the DEGs were characterized using the GO and KEGG databases. Based on recent research results [25, 29, 31, 42, 44], we will focus on gene regulatory network models related to carpel and ovule development, “Plant hormone signal transduction” (ko04075), “Zeatin biosynthesis” (ko00908), “hormone biosynthetic process” (GO:0042446), and “cytokinin biosynthetic process” (GO:0009691) (Additional files 2).

### Gene regulatory network (GRN) models related to carpel and ovule development

**Fig. 8:**
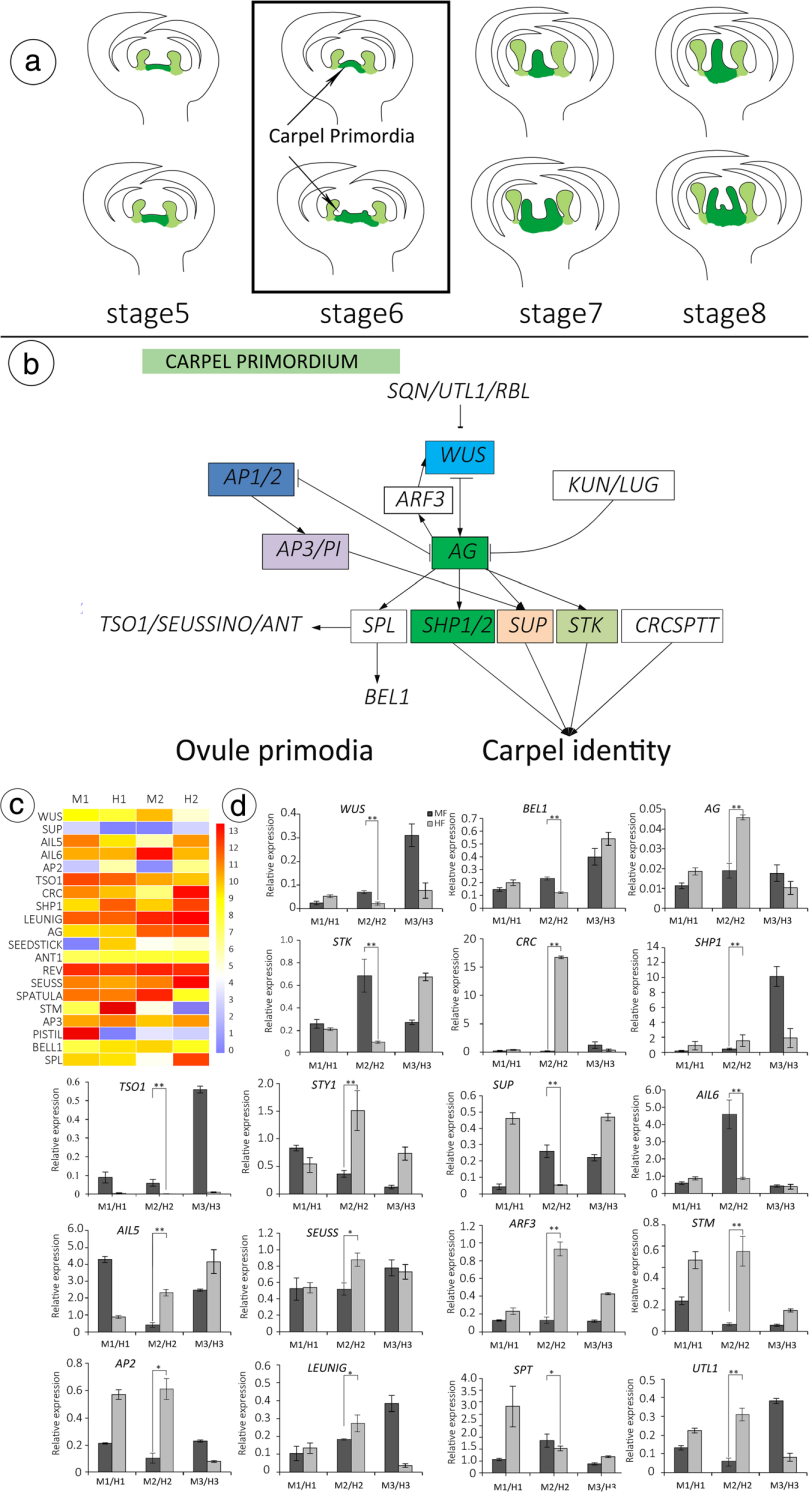
Expression of candidate genes involved in pistil development. **b** Flower development in the MF-HF difference model. **b** Based on prior work in Arabidopsis [23, 58–63], a proposed model of gene-dependent pathways playing potential roles in regulating gynoecium development. **c** Expression of candidate genes involved in ovule development. **d** Relative expression levels of 20 selected genes at different stages of floral bud development in MF and HF. Relative expression levels were calculated by the 2-ΔΔCt method with actin as a standard. 11-Apr., 21-Apr., and 4-May represent the pistils of flowers at stage 5, stage 6, and stage 8, respectively. Different colours from blue to red show the relative log2 (expression ratio)

The results of scanning electron microscopy (SEM) and light microscopy (LM) show that most of the vestigial pistils of MF of *T. sinensis* developed into finger-like structures without ovary or ovule. Pollen germination and pollen tube growth in MF indicated that pollen tubes can grow in the stigma/style and that they are functional, and therefore, ovules are never present is the main factor responsible for female-sterility of male flowers (Fig. 8a). In Arabidopsis, ovule cell fate is controlled by ovule identity genes [31, 44, 58–60]. The pathway of regulation of *Arabidopsis* ovule development is shown in Fig. 7, and key regulators include *AG* [30, 61, 62], *SPL* [63], *INO* [64], *ANT* and *BEL1* [31, 65]. Carpels are the most complex structures within flowers; a GRN (gene regulatory network model) underlies their development in *Arabidopsis* [31], and in *T. sinensis,* the key regulators were identified, including *WUS* (Unigene0010686), *AGAMOUS* (Unigene0020941), *SUP* (Uniene0047137), *SPL* (Unigene0006964), *INO* (Unigene0025624), *STM* (Unigene16944), *ANT1* (Unigene0002499), *STK* (*AGL11*) (Unigene0007536), *SHP1* (Unigene0013614), *AP2* (Unigene0015664), *TSO1* (Ungene0002282), *SPT* (Unigene0034455), *LUG* (Unigene0041412), *PI* (Unigene0020391), *SEUSS* (Unigene0033961) and *BEL1* (Unigene0039261) (Fig. 8b, c). In the MF-HF comparison, these 16 genes showed significantly different expression levels. The results indicated that these genes may influence the flower development.

To confirm that the unique-match genes from the Illumina sequencing and bioinformatics analysis were indeed differentially expressed, a total of 16 genes were selected from the DEGs related to flower development (including carpel, ovule, pollen) for quantitative RT-PCR assays. For 16 genes, the qRT-PCR analysis revealed the same expression trends as the RNA-Seq data, despite some quantitative differences. All genes had different expression levels between MF and HF at stage 6 (Fig. 8d). For example, at stage 6, Unigene0020941, a transcription factor that regulated carpel development, was down-regulated (0.018 to 0.045) in MF relative to HF by qRT-PCR, the same trend as the one (39 to 338) from RNA-Seq.

### Identification of genes involved in ethylene biosynthesis and signal transduction

**Fig. 9:**
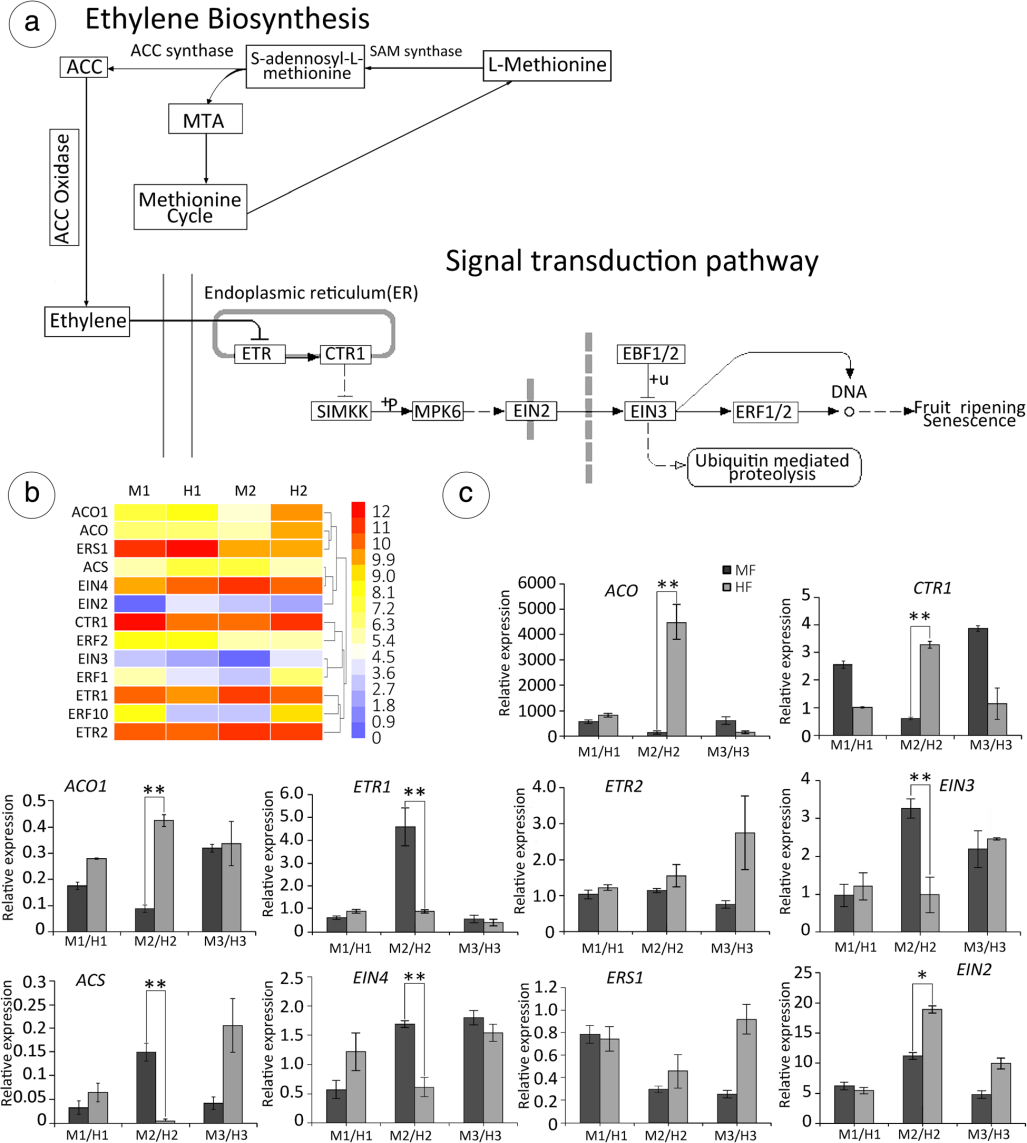
Expression of candidate genes involved in ovule development. **a** Ethylene biosynthesis and signal transduction pathway, which comes from a KEGG pathway enrichment analysis [76, 77]. **b** Expression of candidate genes involved in ethylene biosynthesis and signal transduction. Gene expression was measured by log2Ratio. 11-Apr, 21-Apr, 4-May, 11-May and 21-May represent the pistils of flowers when their development stages were 5, 6, 10, 12 and 14, respectively. However, here, we mainly discussed stage 6, which is the most important development stage in sex differentiation in T. sinensis (for details, see ‘Structure of the Flower’). Different colours from blue to red show the size of the correlation coefficient. **c** Relative expression levels of 10 selected genes at different stages of floral bud development in MF and HF. Relative expression levels were calculated by the 2-ΔΔCt method with actin as a standard. * represents the *P* < 0.05 level of significant difference, and ** represents the *P* < 0.01 level of significant difference in independent-samples *t*-tests

Ethylene regulates many aspects of plant development and responses to biotic and abiotic stress. We identified ten DEGs involved in the ethylene biosynthesis and signal transduction process (Fig. 9a): *ACO* (Unigene0029908), *ACO1* (Unigene0037768), *ACS* (Unigene0021193), *ERS1* (Unigene0024060), *CTR1* (Unigene0036282), *TIR1* (Unigene0037222), *ETR1*/*2* (Unigene0020715, Unigene0027393), *EIN2/3/4* (Unigene0029458, Unigene0050718, Unigene0003076) (Fig. 9b). To confirm the results of RNA sequencing, ten DEGs related to carpel development were selected for qRT-PCR analysis (Fig. 9c). The results showed similar expression trends to the transcriptome analysis, and only 2/16 genes (*EIN2* and *EIN3*) had different results in qRT-PCR vs RNA-Seq, confirming the reliability of the RNA-Seq data. 1-aminocyclopropane-1-carboxylic acid (ACC) synthase (ACS) is the rate-limiting enzyme in ethylene synthesis, and it is important in regulating ethylene biosynthesis. Unigene0021193 was downregulated at stage 6 in the M-H comparison. Two *ACC* oxidase homologous genes showed different expression patterns, but *ACO* and *ACO1* were both upregulated in stage 6 in the MF-HF comparison.

In *Arabidopsis*, the perception of ethylene is achieved by five members of a family of ER membrane-bound receptors: *ETR1*, *ETR2*, *ERS1*, *ERS2* and *EIN4*, some of which have histidine kinase activity [72]. From mRNA sequencing, we obtained four ethylene receptors: *EIN4* (Unigene0003076), *ETR2* (Unigene0027393), *ETR1* (Unigene0020715), *ERS1* (Unigene0024060). At stage 6, the relative expression of Unigene0020715 displayed significant differences, but Unigene0027393 and Unigene0024060 were indistinctive. Unigene0020715, the repressor of ethylene signal transduction, was up-regulated in MF relative to HF, but Unigene0027393 displayed indistinctive expression. Similarly, Unigene0003076 displayed almost the same trend as *ETR1* during stage 6*,* while Unigene0024060 was indistinctive. *ERF1/2* (Unigene0025860, Unigene0005688), the downstream gene in the ethylene signal transduction pathway, displayed differential expression in both the M1-M2 and H1-H2 comparisons, being up-regulated in MF relative to HF at the key stage. Obviously, at stage 6, *ACO* (Unigene0029908), *ACO1* (Unigene0037768), *CTR1* (Unigene0036282), and *EIN2* (Unigene0029458) were up-regulated in the MF-HF comparison, while *ACS* and *EIN3* were down-regulated in MF relative to HF.

To verify the role of ethylene in sex differentiation of *T*. *sisensis*, we sprayed ethephon at concentrations of 150 mg/L. We found that ethephon had a significant effect on MF transformed into HF.

### Identification of genes involved in Auxin and Cytokinin signal transduction

**Fig. 10:**
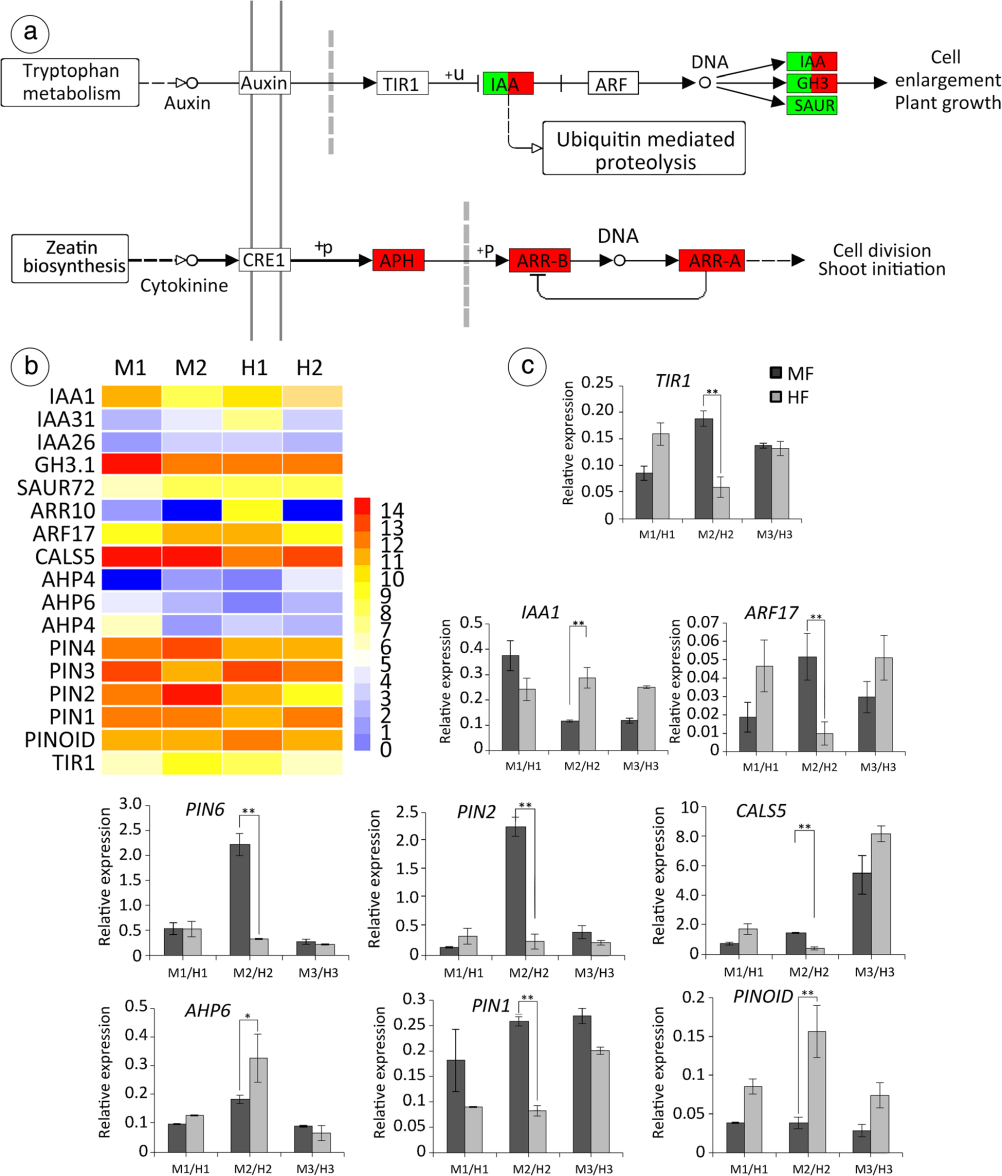
**a** Auxin and cytokinin signal transduction. **b** Expression of candidate genes involved in ethylene biosynthesis and signal transduction based on KEGG pathway enrichment analysis. The gene abundances were calculated and normalized to RPKM (Reads Per kb per Million reads). **c** The relative expression levels of 9 selected genes at different stages of floral bud development in M and H. Relative expression levels were calculated by the 2-ΔΔCt method with actin as a standard. 11-Apr, 21-Apr, and 4-May represent the pistils of flowers when their development stages were 5, 6, and 8, respectively. However, here, we mainly discussed stage 6, which is the most important development stage for sex differentiation in T. sinensis (for details, see ‘Structure of the flower’)

The third related pathway was the auxin transport and signalling pathway. As the first plant hormone studied, auxin has wide-ranging effects on growth and development throughout the plant, including gynoecium and ovule morphogenesis [73–75]. In our study, we identified 24 DEGs involved in auxin and cytokinin signal transduction (Fig. 10a, b): *IAA31* (Unigene0018133), *IAA26* (Unigene0035359), *IAA15* (Unigene0001116)*, IAA19* (Unigene0025767), *GH3.1* (Unigene0015426), *SAUR2* (Unigene0017779), *SAUR14* (Unigene0050945), *SAUR72* (Unigene0005319), *SAUR50* (Unigene0010084), *SAUR12* (Unigene0018267), *SAUR31* (Unigene0049968), *SAUR53* (Unigene0019490), *KUP3* (Unigene0005158)*, MIR397B* (Unigene0025946), *ARR10* (Unigene0049560), *AHP4* (Unigene0004154), *AHP6* (Unigene0011561), *AHP4* (Unigene0004325), *IAA1* (Unigene0001116), *ARF17* (Unigene0029174), *CALS5* (Unigene0019058), *PIN6* (Unigene0020253)*, PIN2* (Unigene0033811), *PIN1* (Unigene0000533). To confirm that the unique-match genes from the Illumina sequencing and bioinformatics analysis were indeed differentially expressed, a total of 9 genes were selected from the DEGs related to flower development for quantitative RT-PCR assays. Among 9 of the 24 genes with different expression levels between MF and HF (Fig. 10c) at stage 6, six genes were up-regulated in MF relative to HF, and three genes were down-regulated.

## Discussion

### The male flowers of *T. sinensis* originated from ancestral hermaphroditic flowers

A variety of hypothesized evolutionary pathways have been proposed for the evolution of dioecy from hermaphroditism, and the production of only bisexual flowers is the ancestral condition in angiosperms [3, 7, 16, 78–83]. Theory predicts that at least two mutations are necessary to evolve separate sexes [11]. One must result in male sterility while the second must result in female sterility [27, 29]. Therefore, we examined whether the androdioecy of *T. sinensis* resulted from transformation from female flowers to restore male function in dioecious plants or was caused by pistil sterility in bisexual flowers.

In *T. sinensis*, male flowers in each natural population possess the characteristic of a vestigial pistil, and the receptive stigma is covered by papillary cells. Although the degree of sterility of pistils is different, the males have the potential to form ovules. In addition, in natural populations, no pollen limitation on the reproduction of *T. sinensis* [84, 85]. Therefore, female flowers evolved from female flowers are unlikely to restore their male function to ensure reproduction [15]. Obviously, it is also unreasonable for MF to evolve a pistil if androdioecy arose from dioecy. In addition, as in *Osmanthus fragrans* L., the MF has a vestigial pistil, also suggesting that males evolved from female-sterile mutants among hermaphrodites [13].

The breeding system and other traits mapped on the phylogenetic tree of *Fraxinus* L. (Oleaceae) show that dioecy has three separate origins; in one instance, dioecy evolved from hermaphroditism via androdioecy following the transition from insect to wind pollination [45, 86]. Dioecy may also evolve in conjunction with the evolution of wind pollination in some previously insect-pollinated species [87, 88]. *T. sinensis* is a wind-pollinated and insect-pollinated species [52, 53], and this mixed pollination pattern may be a possible evolutionary pathway from hermaphroditism to dioecy [55]. A trend in tectum sculpture character state change from perforate to reticulate appears to be common in angiosperms [66, 67, 89]. In *T. sinensis*, the tectum sculpture is reticulate in MF pollen grains, while that of HF is perforate. Additionally, globose pollen grains have the largest volume-to-surface area ratio, but with minimal regulatory function, which is a relatively primitive character [68]. Olive pollen grains can be transformed from the globose pollen grains of HF. Evolutionarily, this result also implied that hermaphroditism is more ancestral [69].

### Key regulatory factors of pistil development may be associated with *T. sinensis* sex differentiation

Admittedly, ovule primordia arise from the placenta; however, both ovules and placenta are completely absent in most MFs of *T. sinensis*. Transcriptome analyses showed that sixteen genes related to carpel and ovule development are expressed differentially at the key stage of MF pistil abortion and were verified by qRT-PCR. The molecular mechanisms of ovule development in *Arabidopsis*, cotton, and rice are relatively well known [24–26, 90]. The C-class gene *AG* (*AGAMOUS*) belongs to the MADS-box family, which determines stamen and carpel identity and plays a role in ovule development [31, 44]. The *SHP1* (*SHATTERPROOF 1*), *SHP2* (*SHATTERPROOF 2*), and *STK* (*SEEDSTICK*) genes together with the *SEP* (*SEPALLATA*) genes have overlapping expression patterns with *AG* in an *AG*-independent manner [31]. *ANT* (*AINTEGUMENTA*), *HLL* (*HUELLENLOS*), *SIN2 (SHORT INTEGUMENT 2)*, *INNER NO OUTER* (*INO*), and *SUPERMAN* (*SUP*) as regulators of ovule outgrowth [31, 76, 90, 91]. *AG* is necessary for the determination and termination of floral meristem activity. In the *ag* mutant, delayed floral meristem activity results in the stamens and carpels being replaced by flowers [61]. *WUSCHEL* (*WUS*), which specifies stem cell identity, and *AG* play crucial roles in regulating the initiation or termination of floral meristems [92, 93].

Previous studies have indicated that *SPL/NOZZLE* is required for the initiation of sporogenesis and plays a central role in nucellus formation by antagonizing the expression of *BELL*, *ANT* and *INNER NO OUTER* [94]. Here, four genes are shown to be differentially expressed in MF relative to HF at stage 6. Moreover, *SPL* represses the expression of *ANT* and *INO* to control ovule primordium formation [95]. *SPL* was downregulated in the key stage of pistil abortion in MF relative to HF, which indicated the role of *SPL* in promoting ovule development. However, during ovule development, *BEL1* and *SPL* are required for cytokinin and auxin signalling for the correct patterning of the ovule [96].

In addition, the *STM* gene is considered to play a pivotal role in sustaining stem cell function in the floral apical meristem (*STM* is required for maintenance of *WUS* expression but independent of *WUS* expression) and controls *KNOX* gene expression independently of the transcriptional repressor *AS1* [97, 98]. More precisely, *STM* plays a crucial role in preventing meristem cell differentiation by inducing the production of cytokinins (CK) and the ARR transduction pathway [31]. The progressive loss of *STM* causes floral phenotypes ranging from reduced formation of placental tissues and inhibited carpel fusion to complete loss of carpel development [99]. In *T. sinensis*, *STM* also showed differential expression, which indicated the role of STM in promoting ovule development.

In *Arabidopsis*, the ovule primordia arise from the placentas flanking the medial ridges, while *leunig* (*lug*) *aintegumenta* (*ant*) double mutants lack medial tissues, and the double mutant *seu*-3 *ant*-1 results in a complete loss of ovule initiation, caused by severe defects in early gynoecium development [100]. *CRABS CLAW* (*CRC*) is a member of the YABBY gene family, whose expression is largely limited to carpels and nectaries, and plants expressing 35SCaMV::CRC produce variable flowers with partial transformations of sepals into carpels and reductions in flower organ number. Carpels are short and solid (ovule is completely absent) and composed primarily of stylar tissues. The morphology of the pistillode of MF in *T. sinensis* was similar to that of the *Arabidopsis* 35SCaMV::CRC pistil [71].

### Ethylene may act as an upstream factor for female sterility in male flowers

Ethylene, as an upstream factor for pistil or ovule formation, has been verified in many species; for instance, tobacco, pomegranate, and cucumber [42, 101, 102].

The ETR1 protein acts as negative regulator of ethylene responses [103], has the function of promoting cell elongation. In *T. sinensis*, *ETR1* (Unigene0020715) was up-regulated in MF relative to HF. *ERF1* and *ERF2*, as ethylene response signal factors, were differentially expressed between MF and HF. The down-regulation of ERF1/2 and up-regulation of *ETR1* may be related to the flower development. ACO and ACS (ACS and ACO are rate-limiting enzyme) are a pivotal enzyme in ethylene biosynthesis, they are showed significant expression levels in pistil formation ant stage 6. Jointly, our findings indicated that ethylene may act as one of the upstream regulators influencing the development of pistil. Furthermore, *ANT*, an *APETALAP2*-like Gene, plays fundamental roles in ovule formation and megasporogenesis [38, 104, 105], the down-regulation of *ANT* may be responded to ethylene. However, the expression of *ANT* and *INO* (*INNER NO OUTER*) may be regulated by *AG* and *SPL*.

### Auxin and Cytokinin are required for ovule Primordia formation

Auxin maxima are fundamental for the formation of primordia, and auxin action has been well described for lateral roots and primordial flowers [44, 106, 107]. The directionality of the auxin flux depends principally on the polar localization of the PIN proteins. In *Arabidopsis*, there are eight PIN proteins (*PIN1*–*8*), from which only *PIN1* and *PIN3* are expressed in the pistil and ovules. For example, polar auxin transport is mostly mediated by the *PINFORMED1* (*PIN1*) efflux carriers [83]. Based on the phenotypes of *pin* mutant, gynoecia display a range of phenotypes from almost normal structures with two valves, style and stigma, to stalk-like gynoecia with an elongated gynophore topped with a style and stigma [108]. The protein kinase *PINOID* (*PID*) positively regulates auxin efflux, which can control an early general step in meristem development. In the *pid-l* flower, the internal fourth whorl organ is stem-like rather than carpelloid, although it is capped with a ring of stigmatic papillae. Furthermore, the morphology of the vestigial pistil of MF *T. sinensis* was similar to that of the *Arabidopsis pid-l* pistil [109]. Similarly, *PINOID* is required for formation of the stigma and style in rice [110]. In addition, the crosstalk between CK and auxin plays a pivotal role in the boundaries in the inflorescence meristem and ovule primordia formation [44]. Simultaneously, the *Arabidopsis* Histidine Phosphotransfer Protein 6 (AHP6), an inhibitor, mediates an auxin-cytokinin crosstalk that regulates the timing of organ initiation at the shoot apical meristem. In this study, genes that were expressed differently in MF relative to HF also supported this point.

## Conclusion

**Fig. 11:**
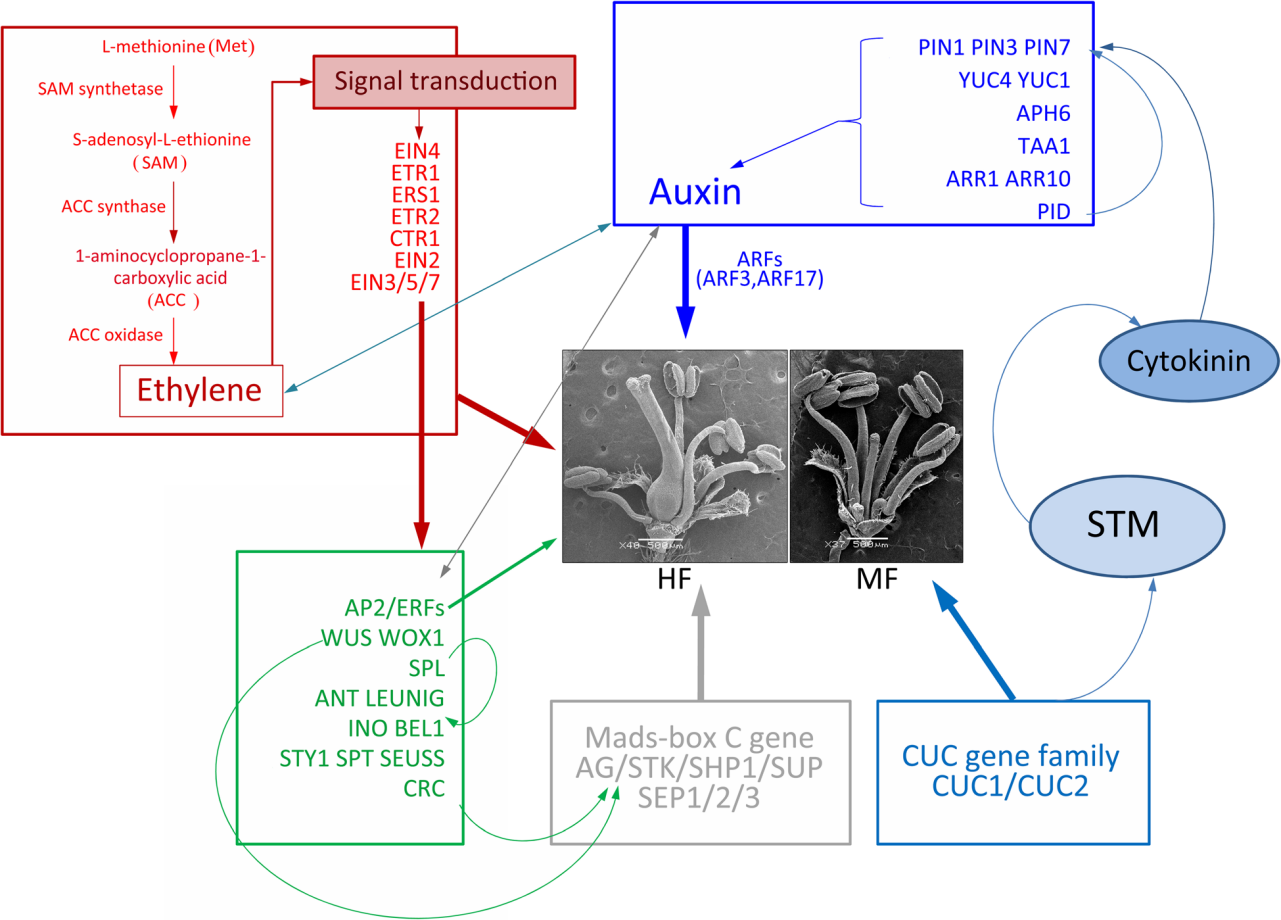
A proposed model of ethylene, auxin, identity genes, and CUC gene family-dependent pathways for gynoecium development. All genes displayed in this figure correspond to DEGs in this study; other related genes without differences are not shown here. Different colours of rectangles represent different pathways or factors; red: ethylene synthesis and metabolism; blue: polar auxin transport; light blue: CUC gene family; grey: MADS-box C genes. Arrows and bars indicate positive and negative regulatory interactions, respectively. These regulatory networks come from the relationships determined in Arabidopsis [31, 44, 60, 105, 118–122]

In total, 29,585 out of 52,945 unigenes were annotated, and the KEGG enrichment analysis will provide valuable information on the sex differentiation of *T. sinensis*. First, ethylene is an upstream factor in *T. sinensis* gynoecium development, and signal transduction of auxin and cytokinin is fundamental to the initiation of pistil primordia; MADS-box genes were also shown to be involved in the determination of the fourth-whorl flower organs. In addition, auxin signalling pathways were thought to be involved in pollen abortion (partial pollen abortion in *T. sinensis* HF populations; Fig. 5) and were important for megagametophyte development (male mutant-Type III in MF; Fig. 1). Overall, a valuable new model of sex differentiation in *T. sinensis* was summarized, and further study of the functions of sex-related genes will be helpful in elucidating the sex-determination mechanism of the androdioecious plant *T. sinensis* (Fig. 11).

## Methods

### Flower sample collection

*T. sinensis* in natural populations has 2 sexual types: male individuals (MF, male flower) and hermaphroditic individuals (HF, hermaphroditic flower) with winter fruits. Two types of flowers of *T. sinensis* were sampled from the natural geographical distribution in Shennongjia National Nature Reserve (31°38′N, 110°25′E; Hubei, China) and the voucher specimens were deposited archived in the herbarium of Structural Botany Laboratory (SBL), Northwest University. Prof. Liu Wenzhe undertook the formal identification of the samples. Different growth stages of flowers were collected for our study. The flowers used for microscopic observation were fixed immediately in 2.5% glutaraldehyde, and those used for transcriptome analysis were stored in liquid nitrogen (LN) at − 80 °C.

A total of 18 samples were sequenced, comprising males and hermaphrodites in three different development stages (with three biological replicates for each stage). To describe the samples clearly, we used M1, M2, and M3 to represent MF buds when their development was at stage 5, stage 6, and stage 8, respectively (Fig. 1a-e). Stages are numbered as described by Sanders et al. (1999) [111]. Three stages of samples were collected mainly according to the results of anatomy (Figs. 3d, e and 4); these stages represent the key stages that implied the formation of sexual dimorphism in *T. sinensis*. Similarly, H1, H2, and H3 represented the HF buds when their development was at stage 5, stage 6, and stage 8, respectively. Photographs are included showing the general developmental stages of the flowers (Fig. 1a-e).

### RNA sequencing library construction, Illumina sequencing, and data processing

Total RNA (40 μg) was isolated from the M1, M2, M3 and H1, H2, and H3 samples using the TRIzol Kit (Promega, Beijing, China) and treated with RNase-free DNase (Takara Biotech Incorporation, Otsu, Japan). RNA quantity and purity were assessed using a Nanodrop 1000 spectrophotometer (Thermo Scientific, Waltham, MA, USA). RNA integrity (> 8.0) was determined using a Bioanalyzer 2100 (Agilent) with an RNA 6000 Nanochip (Agilent). Methods of enrich Mrna, remove rRNA, RNA fragment, random haxamer primed cDNA synthesis, size selection and PCR amplification were performed as previously described [43, 112, 113], and sequenced using Illumina HiSeqTM 4000 (Gene Denovo Biotechnology Co., Guangzhou, China).

### De novo assembly and functional annotation

Raw data were processed with Perl scripts to ensure the quality of the data used in further analyses. The adopted filtering criteria are as Zhang et al. (2018) [55, 114]. The obtained clean data after filtering were used for statistical analyses of quality, including Q30, data quantity and quality, and base content statistics. The software Trinity-v2.6.5 was used for de novo assembly [55]. TransDecoder identifies candidate coding regions within transcript sequences, such as those generated by de novo RNA-Seq transcripts assembled by Trinity. Functional annotation of transcript assemblies was performed using the included Trinotate pipeline (http://trinotate.sourceforge.net/) to identify open reading frames and assign best hits to UniProtKB (1e-03), PFAM-A (1e-03), Gene Ontology (GO) and eggNOG categories.

### Screening of differentially expressed genes

To identify DEGs across samples or groups, the edgeR package (http://www.r-project.org/) was used. We identified genes with greater than a two-fold change and a false discovery rate (FDR) < 0.05 in a comparison as significant DEGs. DEGs were then subjected to an enrichment analysis of GO functions and KEGG pathways. Read count for each gene in each sample was determined by HTSeq (v0.6.0; http://www-huber.embl.de/users/anders/HTSeq/doc/overview.html), and RPKM (reads per kilobase per million mapped reads) was then calculated to estimate the expression level of the genes in each sample [115]. The RPKM method is able to eliminate the influence of different gene lengths and sequencing data amounts on the calculation of gene expression. Therefore, the calculated gene expression can be directly used for comparing differences in gene expression among samples.

Differential expression analysis of the three stages in MF and HF was performed using DESeq [116]. DESeq2 v1.4.5 was used for differential gene expression analysis, and a *p*-value could be assigned to each gene and adjusted by Benjamini and Hochberg’s approach for controlling the false discovery rate. Genes with q ≤ 0.05 and |log2_ratio| ≥ 1 were identified as DEGs. Gene expression patterns in all samples were compared with expression profiles for known *Arabidopsis*, *Zea* and *Oryza* carpel development or sex-determination genes [31, 58–60, 70, 78, 106, 115].

### GO and pathway enrichment analysis of DEGs

The GO (Gene Ontology; http://geneontology.org/) enrichment of DEGs was implemented by the hypergeometric test, in which a p-value is calculated and adjusted as the q-value, and the data background is genes in the whole genome. GO terms with q < 0.05 were considered to be significantly enriched. GO enrichment analysis could reveal the biological functions of the DEGs [112], and we focused on genes that play roles in certain biological functions, i.e., those for which pathway enrichment analysis (KEGG, Kyoto Encyclopedia of Genes and Genomes) was mainly concerned with metabolic pathways or signal transduction pathways. Pathway analyses were performed with the KEGG pathway database using BLASTx with an E-value < 1 e-5 [117]. The calculating formula is the same as that in GO analysis.

$$ P=1-\sum \limits_{i=0}^{m-1}\frac{\left(\begin{array}{l}M\\ {}i\end{array}\right)\;\left(\begin{array}{l}N-M\\ {}n-i\end{array}\right)}{\left(\begin{array}{l}N\\ {}n\end{array}\right)}\kern0em $$

Here N is the number of all genes that with KEGG annotation, n is the number of DEGs in N, M is the number of all genes annotated to specific pathways, and m is number of DEGs in M. The calculated *p*-value was gone through FDR Correction, taking FDR ≤ 0.05 as a threshold. Pathways meeting this condition were defined as significantly enriched pathways in DEGs.

### Real-time RT-PCR analysis

qRT-PCR was performed not only to analyze the expression levels of DEGs but also to verify the RNA-Seq analysis, which included ten genes (*CTR1*, *ACS*, *ACO*, *ACO1, ETR1*/*2*, *ERS1*, *EIN2/3/4*) related to ethylene biosynthesis and signal transduction, nine genes involved in auxin and cytokinin signal transduction, and sixteen genes related to carpel and ovule formation. Primer sequences were designed using Primer premier 5.0, and Primer sequences and β - actin (as an internal control to normalize the expression levels of genes) sequence of internal reference gene are listed in Additional file 1. RNA was extracted from *T. sinensis* of three developmental stages, and RNA extraction, cDNA synthesis, and Real-time PCR method as described in related references [116, 123].

## Supplementary information

**Additional files 1**: Primer sequences and the results of DEGs

**Additional files 2:** GO analysis in M1_vs_H1, M2_vs_H2, and M3_vs_H3.

**Additional files 3:** GO and KEGG pathway enrichment analysis in TERM1 and TERM2.

**Additional file 4:** **Figure S1**. Differences between mature HF and TYPE III MF. (**A**) Longitudinal section of HF. (**B**) Mature embryo sac in HF. (**C**) In Type III MF, embryo sac development stopped at the triad stage. (**D**) Longitudinal section of Type III MF. Sy, synergid; sn, secondary nucleus; hy, hypostase; ii, inner integument; oi, uter integument; mi, micropyle. **Figure S2**. Eight model profoles in TERM1 and TERM2, respectively. In TERM1, the expression patterns of 13,761 genes were analysed, eight model profiles were used to summarize, and five expression patterns of genes showed significant p-values (*p* < 0.05) (coloured boxes). In TERM 2, the expression patterns of 16,130 genes were analysed, and eight model profiles were used to summarize. Three expression patterns of genes showed significant p-values (*p* < 0.05) (coloured boxes). Each box represents a model expression profile with the model profile number and *p*-value. Colored boxes indicate that there are significant differences between floral stages. No color box means no difference. M1, M2 and M3 represent MF at stages 5, 6 and 10, respectively, while H1, H2 and H3 represent bisexual flowers at stages 5, 6 and 10, respectively. The meaning of the ‘significant *p*-value’ was a significant difference between floral stages. **Figure S3**. Numbers of differentially expressed genes. (**A**) TERM1 – trend all by gene number, trend all by *P*-value. In profile 3: 2364 gene (2.6e-19 *P*-value) had stable expression in floral stages 5 and 6, but decreased in expression in stage 10; in profile 4, 1664 genes (1.1e-11 *P*-value) had stable expression in floral stages 5 and 6, but increased in expression in stage 10; in profile 5, 2320 genes (3.7e-17 *P*-value) increased in expression in floral stages 5 and 6, but decreased in stage 10; in profile 6, 2170 genes (6.2e-06 *P*-value) had an increased expression in floral stages 5 and 6, but had stable expression in stage 10; in profile 7: 1471 genes (7.7e-18 *P*-value) had an increased expression in floral stages 5 to 10. (**B**) TERM2 – trend all by gene number, trend all by P-value. In profile 1, the expression of 2308 genes (3.6e-47 *P*-value) present the trend of first decline and then maintain stable from stage 5 to 10; profile 6: 3179 genes (1.2e-79 *P*-value) had an increased expression in floral stages 5 and 6, but had stable expression in stage 10; profile 7: 1839 genes (5.2e-39 *P*-value), the trend was always upward. (**C**) GO and KEGG pathway enrichment analysis in TERM1 and TERM2. The meaning of the ‘significant *p*-value’ was a significant difference between floral stages.

## Data Availability

The datasets used and/or analyzed during the current study has been included within supplemental data. The plant materials are available from the corresponding author on reasonable request.

## Abbreviations

*ACO*: *AMINOCYCLOPROPANE-1-CARBOXYLATE OXIDASE*
*ACS*: *1-AMINOCYCLOPROPANE-1-CARBOXYLIC ACID SYNTHASE*
*AG*: *AGAMOUS*
*AHP4/6*: *HPT PHOSPHOTRANSMITTER 4/6*
*ANT*: *AINTEGUMENTA*
*AP*: *APETALA 2*
*BEL1*: *BELL1*
CRC: *CRABS CLAW*
*CTR1*: *COPPER TRANSPORT PROTEIN 1*
*CUC1/2*: *CUP*-*SHAPED COTYLEDON 1/2*
DEGs: differentially expressed genes
*EIN2*/*3*/*4*: *ETHYLENE INSENSITIVE PROTEIN 2/3/4*
*ERS1*: *ETHYLENE RESPONSE SENSOR 1*
*ETR1/2*: *ETHYLENE RECEPTOR 1/2*
HF: Hermaphrodite flowers
*IAA*: *AUXIN/INDOLE*-*3*-*ACETIC ACID*
*INO*: *NNER NO OUTER*
*KUP3/50/72*: *K+ UPTAKE TRANSPORTER 3/50/72*
*LUG*: *LEUNIG*
MF: male flowers
OTD: ovary transverse diameters
*PI*: *PISTILLATA*
*PIN*: *PINOID*
*PIN1*: *PINFORMED1*
PL: Pistil lengths
*SAUR*: *SMALL AUXIN UPREGULATED*
SEM: scanning electron microscope
*SEU*: *SEUSS*
*SHP1/2*: *SHATTERPROOF1/2*
*SPL*/*NZZ*: *SPOROCYTELESS*
*SPT*: *SPATULA*
*STK*: *SEEDSTICK*
*STY1*: *STYLISH1*
*SUP*: *SUPERMAN*
*WUS*: *WUSCHEL*
