# Supplementary figures and images for: Anatomy and RNA-Seq reveal important gene pathways regulating sex differentiation in a functionally Androdioecious tree, Tapiscia sinensis
Source: BMC Plant Biol. 2019 Dec 16;19:554. doi: 10.1186/s12870-019-2081-7 (PMC6915933; doi:10.1186/s12870-019-2081-7)

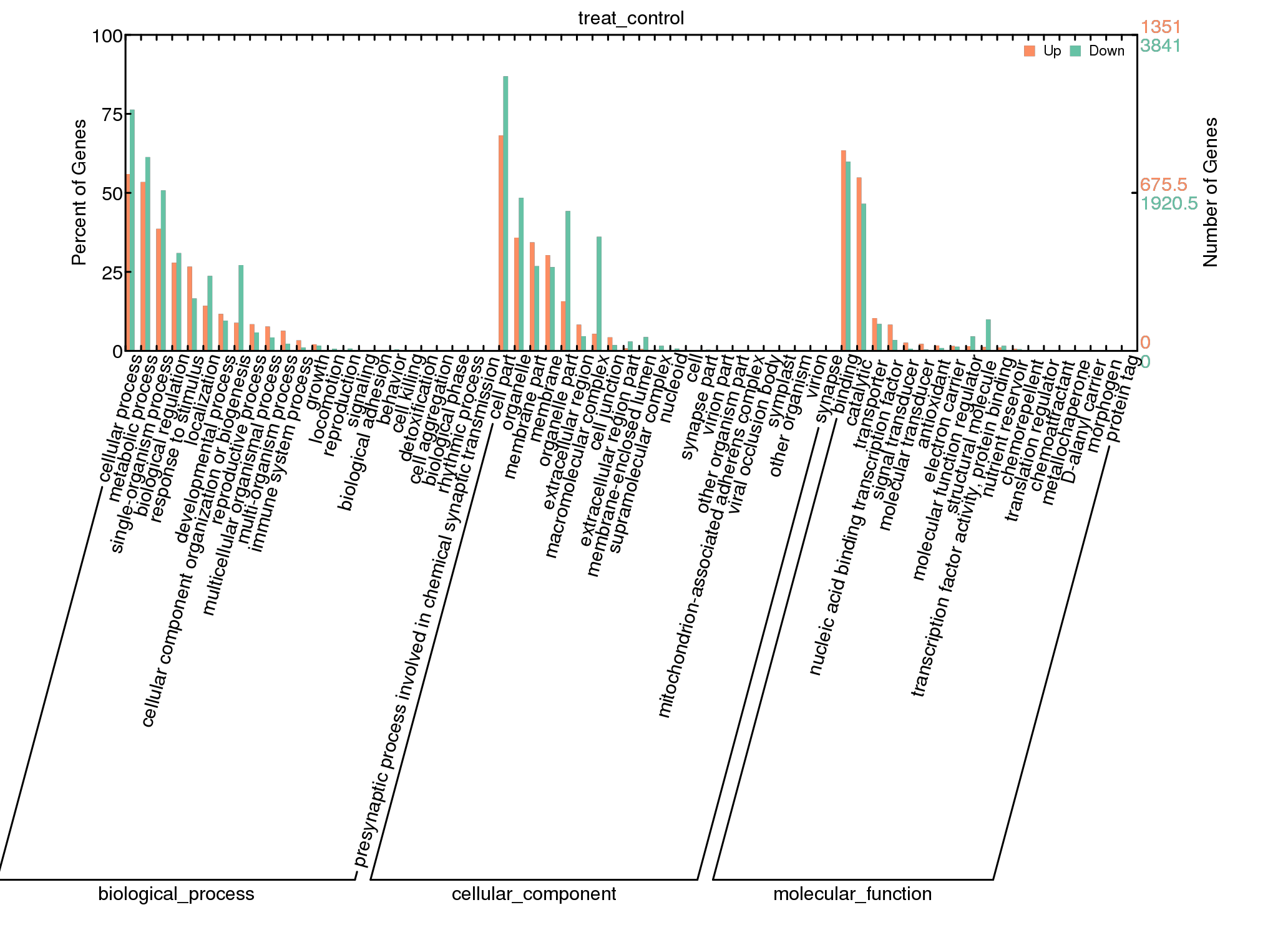

Supplement: Supplementary file 2 — Additional files 2: GO analysis in M1_vs_H1, M2_vs_H2, and M3_vs_H3. [file 12870_2019_2081_MOESM2_ESM.zip › S2 M1-vs-H1_Up_Down.png]

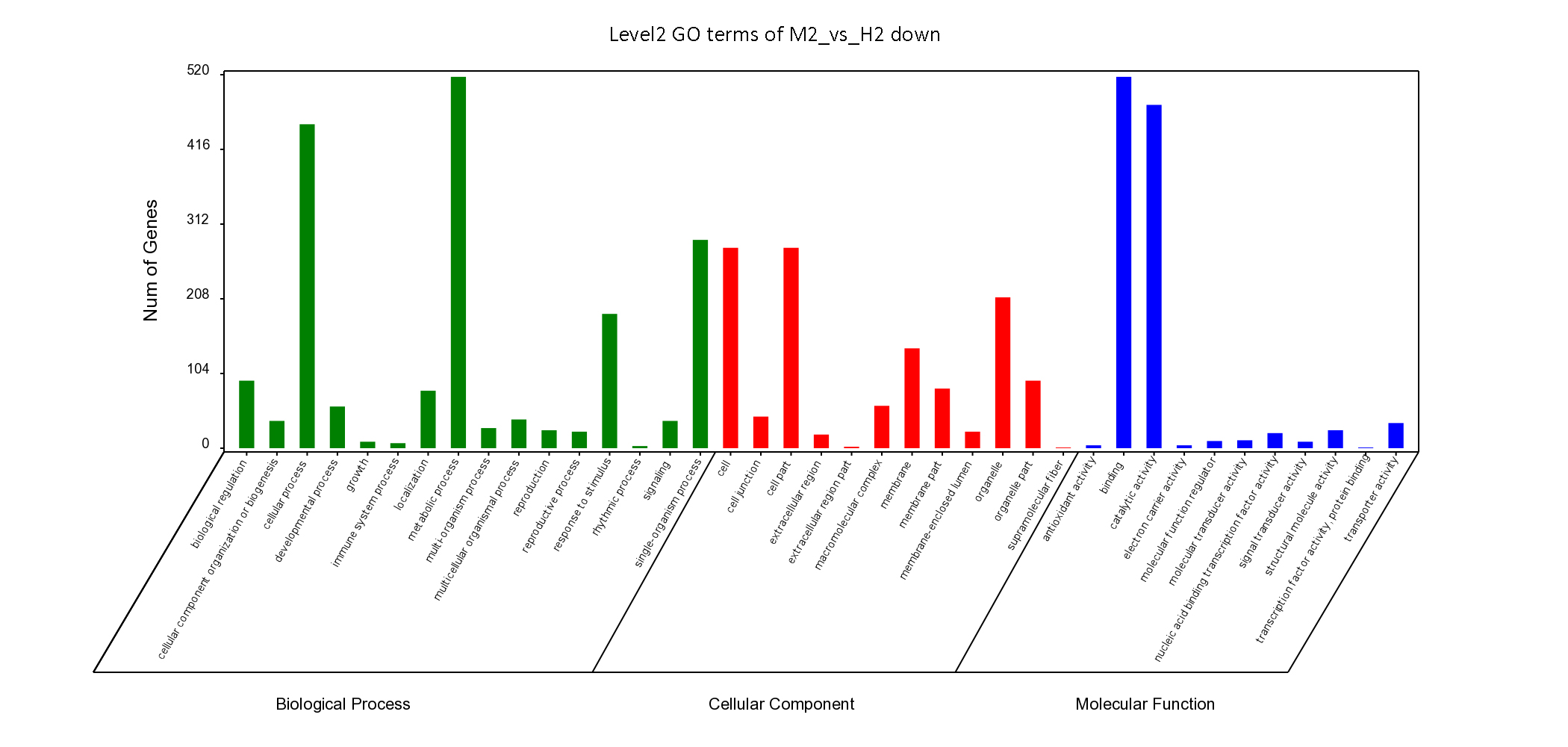

Supplement: Supplementary file 2 — Additional files 2: GO analysis in M1_vs_H1, M2_vs_H2, and M3_vs_H3. [file 12870_2019_2081_MOESM2_ESM.zip › S2 M2-VS-H2_down.Level2.jpg]

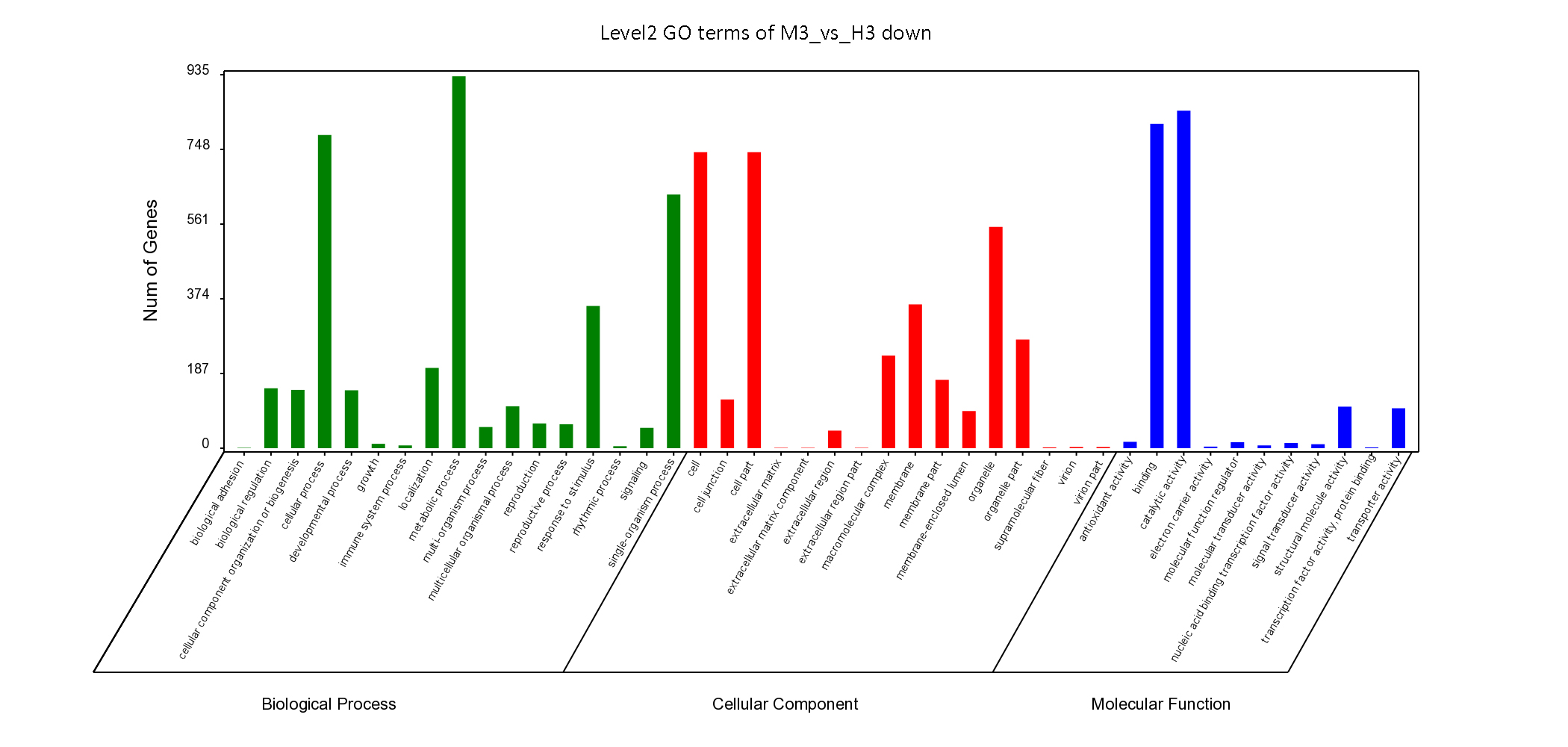

Supplement: Supplementary file 2 — Additional files 2: GO analysis in M1_vs_H1, M2_vs_H2, and M3_vs_H3. [file 12870_2019_2081_MOESM2_ESM.zip › S2 M3-VS-H3_down.Level2.jpg]

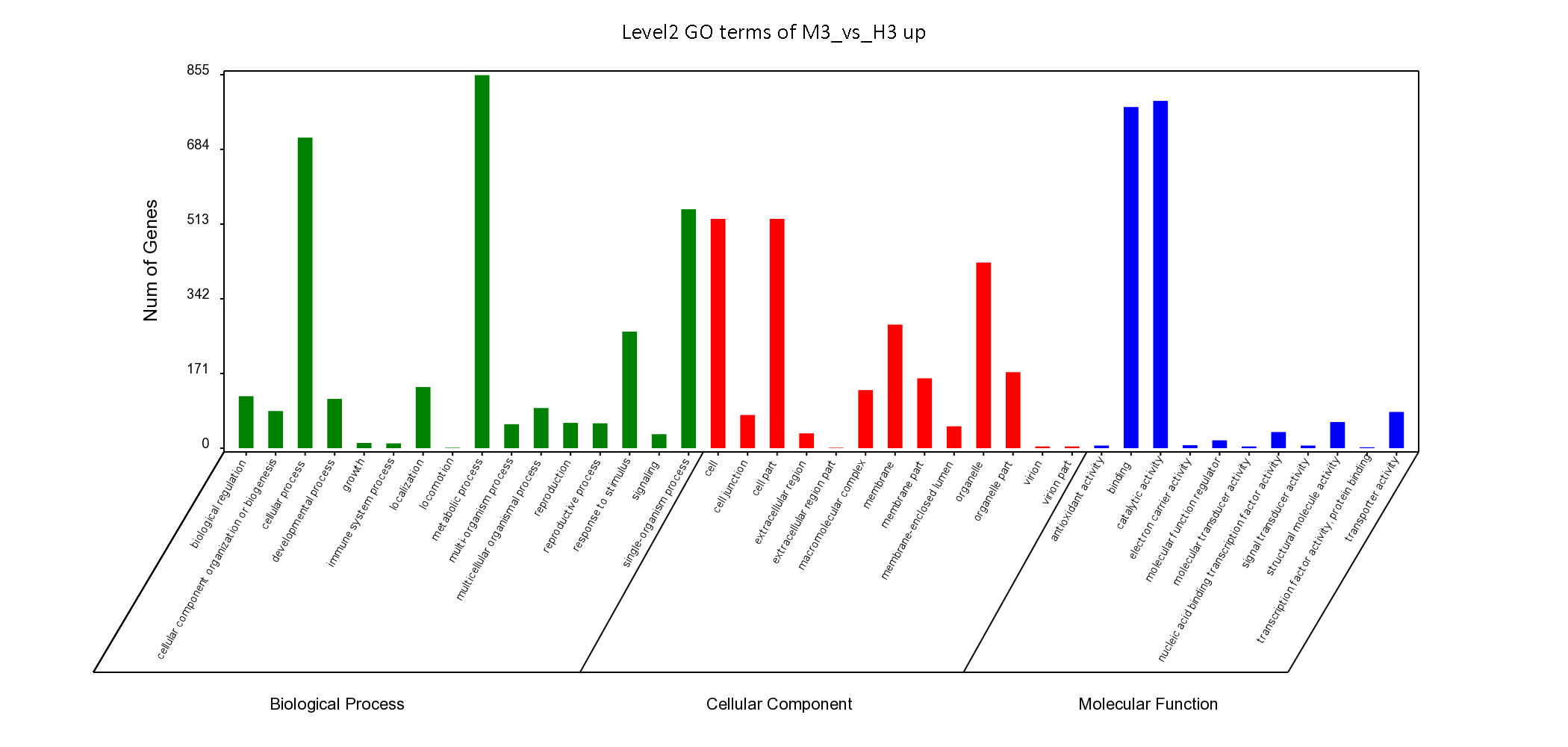

Supplement: Supplementary file 2 — Additional files 2: GO analysis in M1_vs_H1, M2_vs_H2, and M3_vs_H3. [file 12870_2019_2081_MOESM2_ESM.zip › S2 M3-VS-H3_up.Level2.jpg]

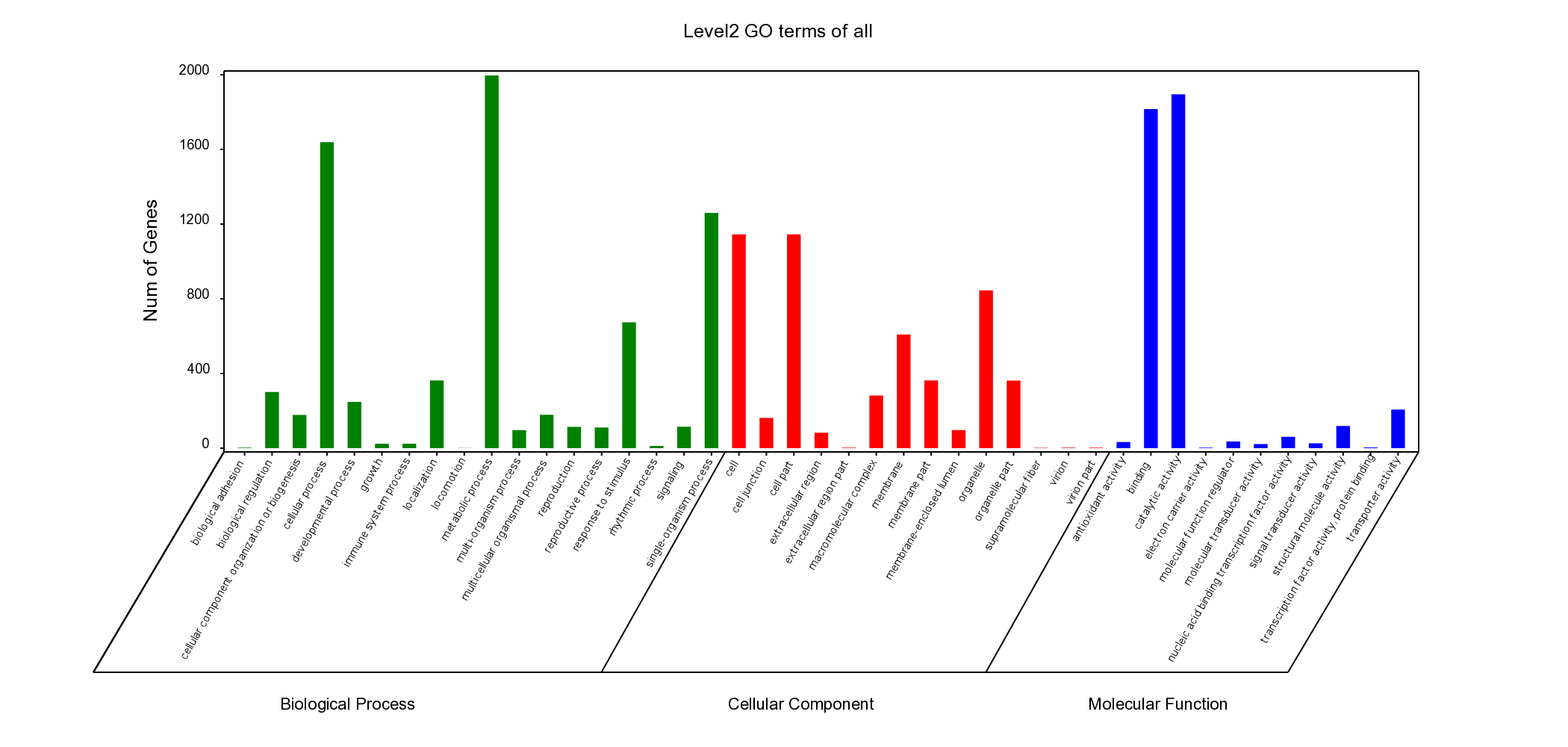

Supplement: Supplementary file 3 — Additional files 3: GO and KEGG pathway enrichment analysis in TERM1 and TERM2. [file 12870_2019_2081_MOESM3_ESM.zip › GO TERM1.png]

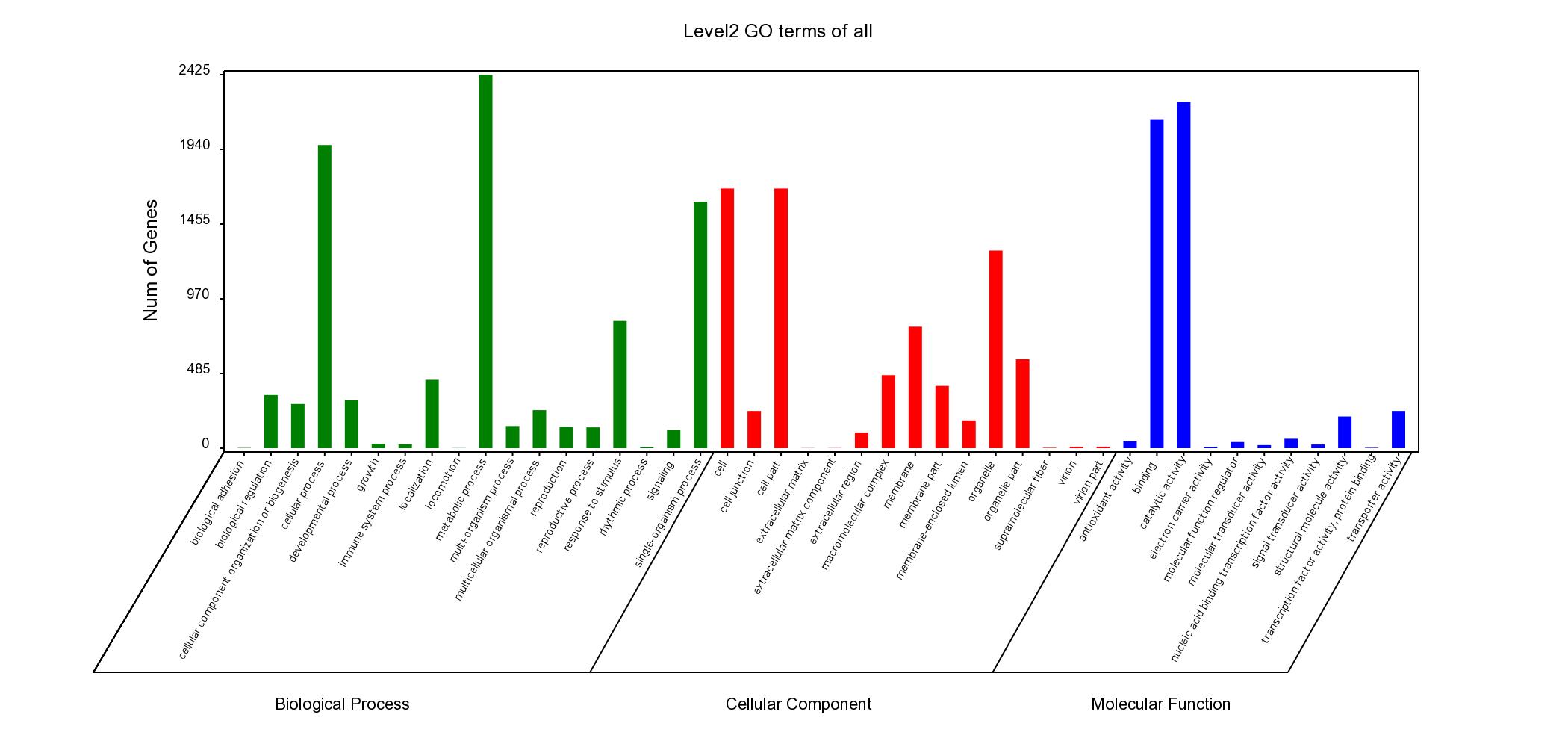

Supplement: Supplementary file 3 — Additional files 3: GO and KEGG pathway enrichment analysis in TERM1 and TERM2. [file 12870_2019_2081_MOESM3_ESM.zip › GO TERM2.png]

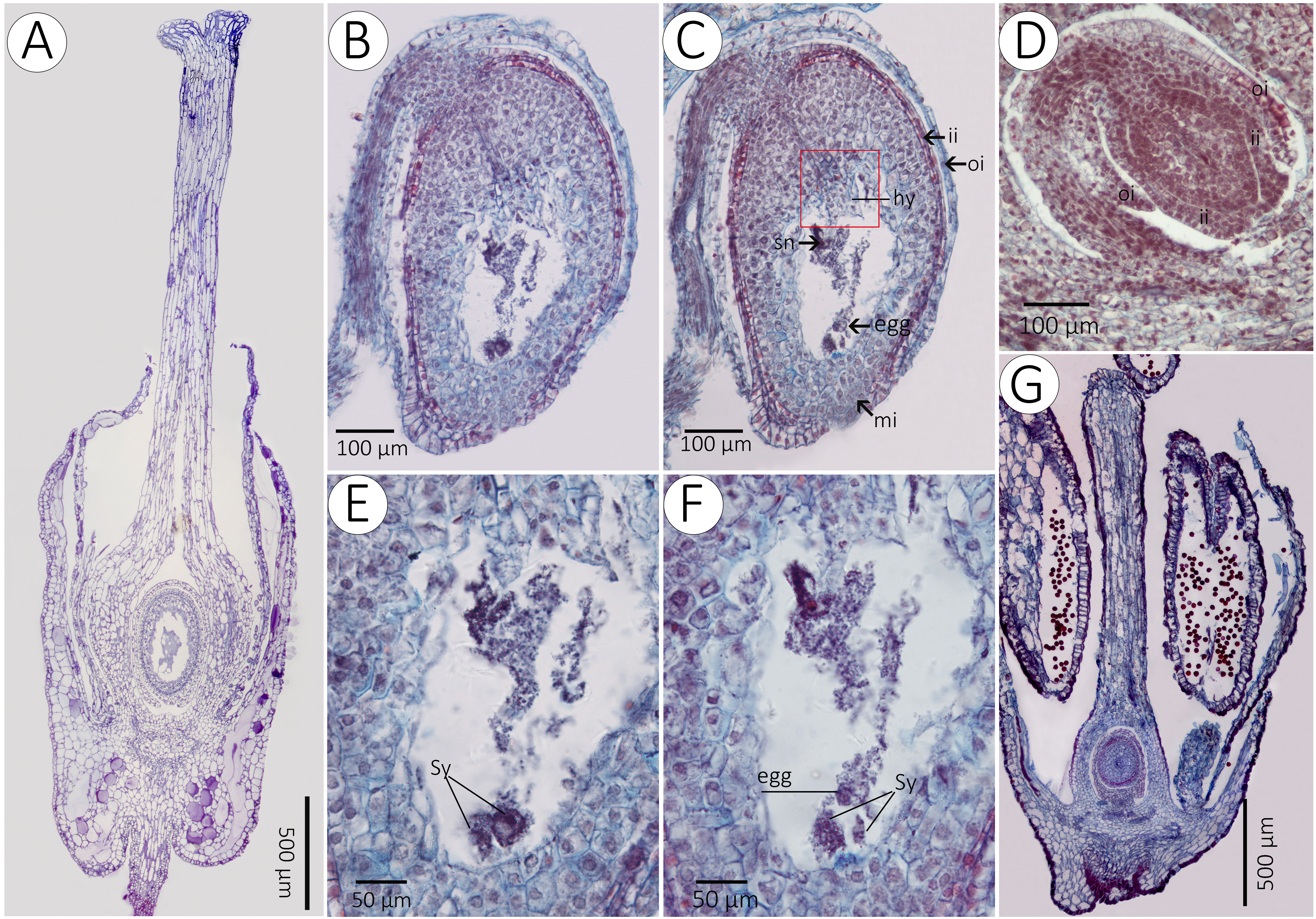

Supplement: Supplementary file 4 — Additional file 4: Figure S1. Differences between mature HF and TYPE III MF. (A) Longitudinal section of HF. (B) Mature embryo sac in HF. (C) In Type III MF, embryo sac development stopped at the triad stage. (D) Longitudinal section of Type III MF. Sy, synergid; sn, secondary nucleus; hy, hypostase; ii, inner integument; oi, uter integument; mi, micropyle. Figure S2. Eight model profoles in TERM1 and TERM2, respectively. In TERM1, the expression patterns of 13,761 genes were analysed, eight model profiles were used to summarize, and five expression patterns of genes showed significant p-values (p < 0.05) (coloured boxes). In TERM 2, the expression patterns of 16,130 genes were analysed, and eight model profiles were used to summarize. Three expression patterns of genes showed significant p-values (p < 0.05) (coloured boxes). Each box represents a model expression profile with the model profile number and p-value. Colored boxes indicate that there are significant differences between floral stages. No color box means no difference. M1, M2 and M3 represent MF at stages 5, 6 and 10, respectively, while H1, H2 and H3 represent bisexual flowers at stages 5, 6 and 10, respectively. The meaning of the ‘significant p-value’ was a significant difference between floral stages. Figure S3. Numbers of differentially expressed genes. (A) TERM1 – trend all by gene number, trend all by P-value. In profile 3: 2364 gene (2.6e-19 P-value) had stable expression in floral stages 5 and 6, but decreased in expression in stage 10; in profile 4, 1664 genes (1.1e-11 P-value) had stable expression in floral stages 5 and 6, but increased in expression in stage 10; in profile 5, 2320 genes (3.7e-17 P-value) increased in expression in floral stages 5 and 6, but decreased in stage 10; in profile 6, 2170 genes (6.2e-06 P-value) had an increased expression in floral stages 5 and 6, but had stable expression in stage 10; in profile 7: 1471 genes (7.7e-18 P-value) had an increased expression in flora [file 12870_2019_2081_MOESM4_ESM.zip › Fig. S1.jpg]

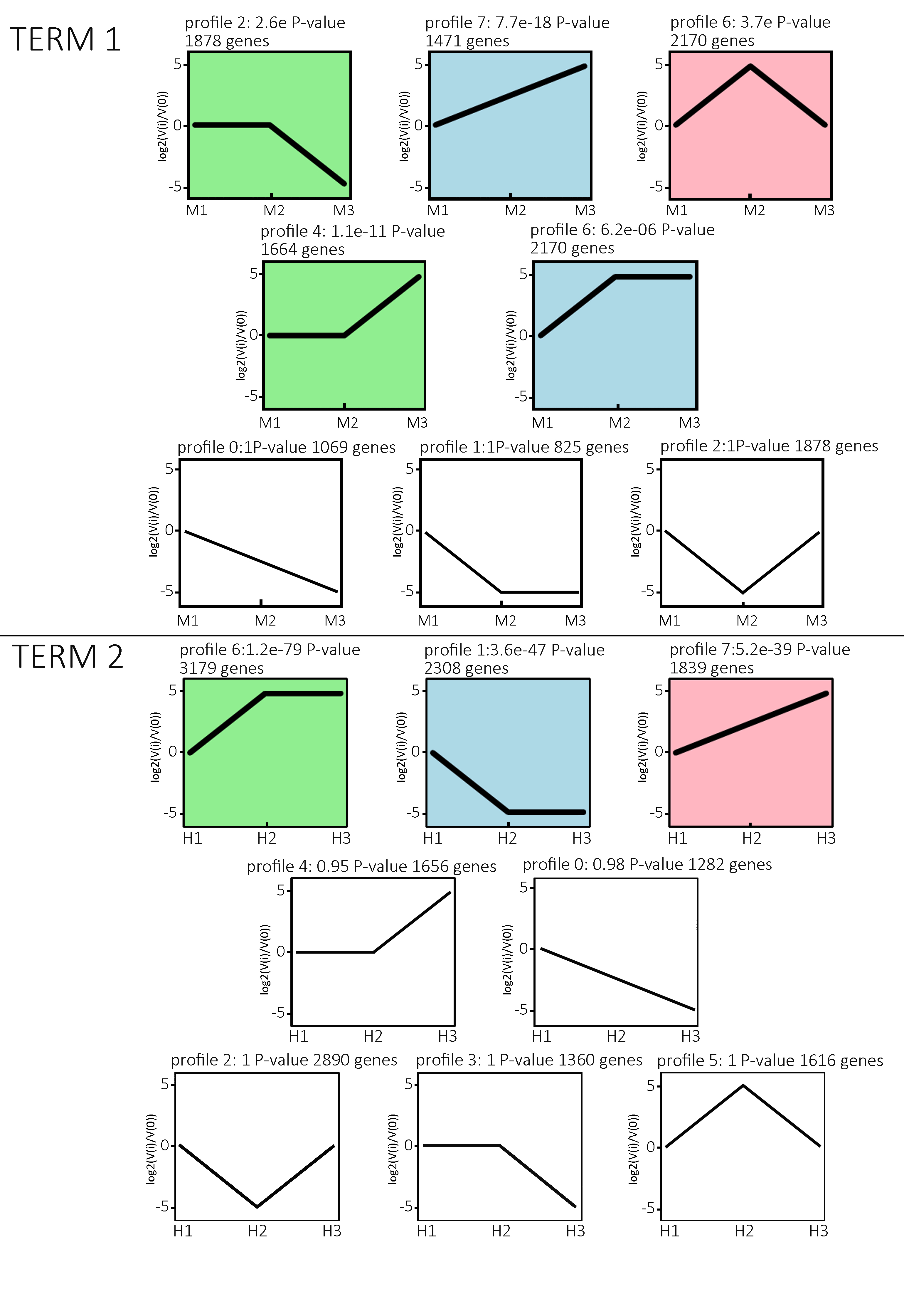

Supplement: Supplementary file 4 — Additional file 4: Figure S1. Differences between mature HF and TYPE III MF. (A) Longitudinal section of HF. (B) Mature embryo sac in HF. (C) In Type III MF, embryo sac development stopped at the triad stage. (D) Longitudinal section of Type III MF. Sy, synergid; sn, secondary nucleus; hy, hypostase; ii, inner integument; oi, uter integument; mi, micropyle. Figure S2. Eight model profoles in TERM1 and TERM2, respectively. In TERM1, the expression patterns of 13,761 genes were analysed, eight model profiles were used to summarize, and five expression patterns of genes showed significant p-values (p < 0.05) (coloured boxes). In TERM 2, the expression patterns of 16,130 genes were analysed, and eight model profiles were used to summarize. Three expression patterns of genes showed significant p-values (p < 0.05) (coloured boxes). Each box represents a model expression profile with the model profile number and p-value. Colored boxes indicate that there are significant differences between floral stages. No color box means no difference. M1, M2 and M3 represent MF at stages 5, 6 and 10, respectively, while H1, H2 and H3 represent bisexual flowers at stages 5, 6 and 10, respectively. The meaning of the ‘significant p-value’ was a significant difference between floral stages. Figure S3. Numbers of differentially expressed genes. (A) TERM1 – trend all by gene number, trend all by P-value. In profile 3: 2364 gene (2.6e-19 P-value) had stable expression in floral stages 5 and 6, but decreased in expression in stage 10; in profile 4, 1664 genes (1.1e-11 P-value) had stable expression in floral stages 5 and 6, but increased in expression in stage 10; in profile 5, 2320 genes (3.7e-17 P-value) increased in expression in floral stages 5 and 6, but decreased in stage 10; in profile 6, 2170 genes (6.2e-06 P-value) had an increased expression in floral stages 5 and 6, but had stable expression in stage 10; in profile 7: 1471 genes (7.7e-18 P-value) had an increased expression in flora [file 12870_2019_2081_MOESM4_ESM.zip › Fig. S2.jpg]

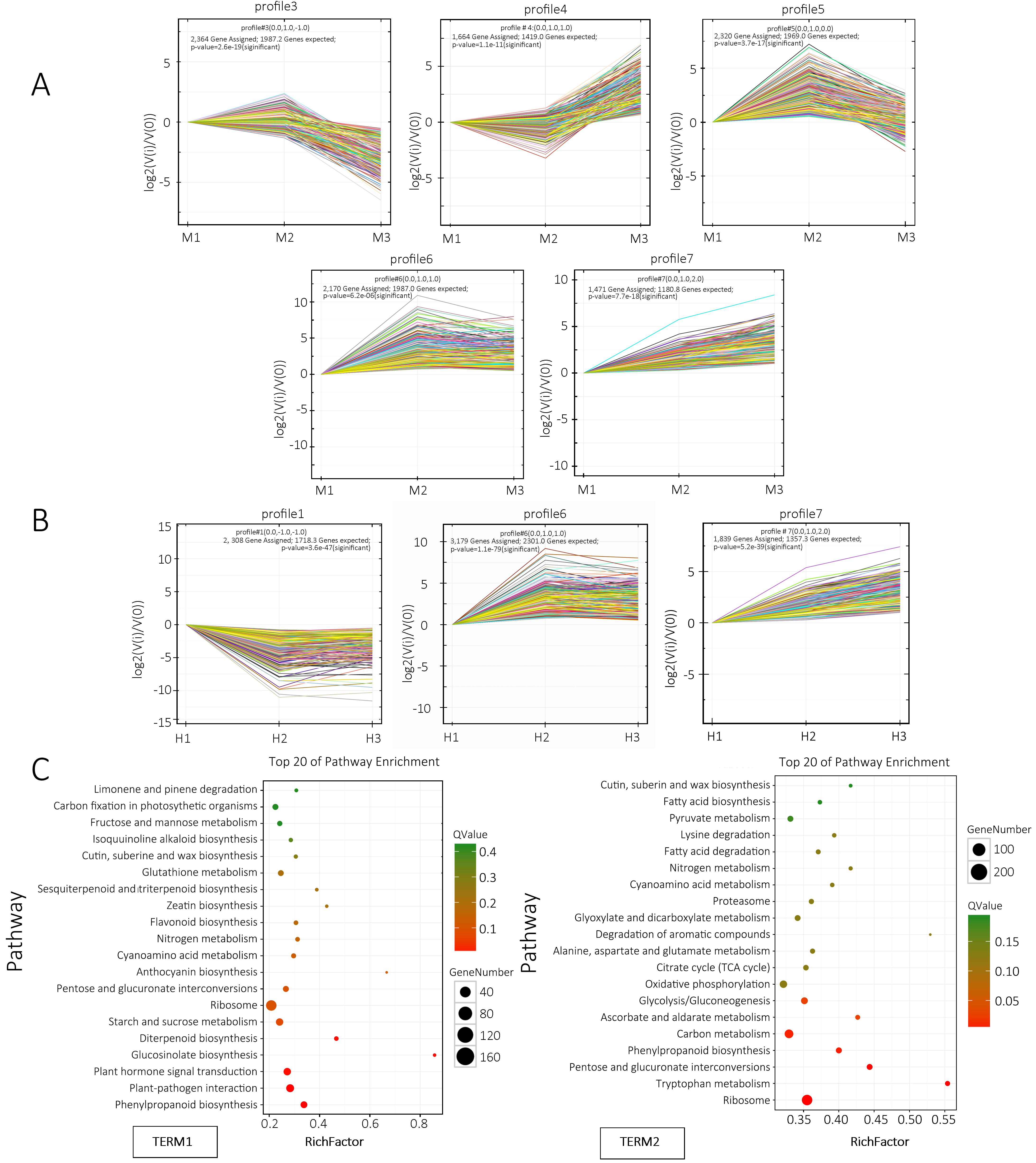

Supplement: Supplementary file 4 — Additional file 4: Figure S1. Differences between mature HF and TYPE III MF. (A) Longitudinal section of HF. (B) Mature embryo sac in HF. (C) In Type III MF, embryo sac development stopped at the triad stage. (D) Longitudinal section of Type III MF. Sy, synergid; sn, secondary nucleus; hy, hypostase; ii, inner integument; oi, uter integument; mi, micropyle. Figure S2. Eight model profoles in TERM1 and TERM2, respectively. In TERM1, the expression patterns of 13,761 genes were analysed, eight model profiles were used to summarize, and five expression patterns of genes showed significant p-values (p < 0.05) (coloured boxes). In TERM 2, the expression patterns of 16,130 genes were analysed, and eight model profiles were used to summarize. Three expression patterns of genes showed significant p-values (p < 0.05) (coloured boxes). Each box represents a model expression profile with the model profile number and p-value. Colored boxes indicate that there are significant differences between floral stages. No color box means no difference. M1, M2 and M3 represent MF at stages 5, 6 and 10, respectively, while H1, H2 and H3 represent bisexual flowers at stages 5, 6 and 10, respectively. The meaning of the ‘significant p-value’ was a significant difference between floral stages. Figure S3. Numbers of differentially expressed genes. (A) TERM1 – trend all by gene number, trend all by P-value. In profile 3: 2364 gene (2.6e-19 P-value) had stable expression in floral stages 5 and 6, but decreased in expression in stage 10; in profile 4, 1664 genes (1.1e-11 P-value) had stable expression in floral stages 5 and 6, but increased in expression in stage 10; in profile 5, 2320 genes (3.7e-17 P-value) increased in expression in floral stages 5 and 6, but decreased in stage 10; in profile 6, 2170 genes (6.2e-06 P-value) had an increased expression in floral stages 5 and 6, but had stable expression in stage 10; in profile 7: 1471 genes (7.7e-18 P-value) had an increased expression in flora [file 12870_2019_2081_MOESM4_ESM.zip › Fig. S3.jpg]

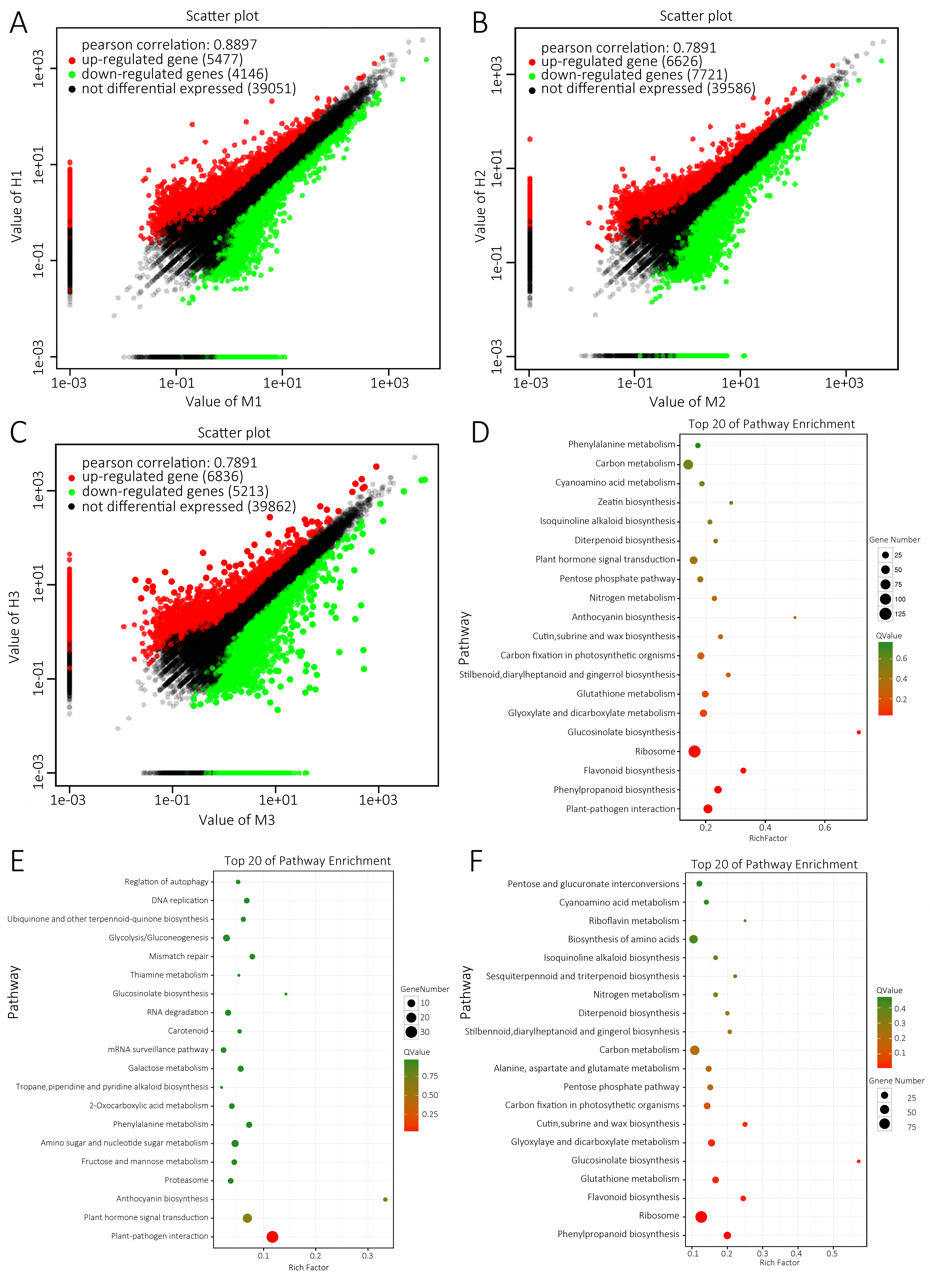

Supplement: Supplementary file 4 — Additional file 4: Figure S1. Differences between mature HF and TYPE III MF. (A) Longitudinal section of HF. (B) Mature embryo sac in HF. (C) In Type III MF, embryo sac development stopped at the triad stage. (D) Longitudinal section of Type III MF. Sy, synergid; sn, secondary nucleus; hy, hypostase; ii, inner integument; oi, uter integument; mi, micropyle. Figure S2. Eight model profoles in TERM1 and TERM2, respectively. In TERM1, the expression patterns of 13,761 genes were analysed, eight model profiles were used to summarize, and five expression patterns of genes showed significant p-values (p < 0.05) (coloured boxes). In TERM 2, the expression patterns of 16,130 genes were analysed, and eight model profiles were used to summarize. Three expression patterns of genes showed significant p-values (p < 0.05) (coloured boxes). Each box represents a model expression profile with the model profile number and p-value. Colored boxes indicate that there are significant differences between floral stages. No color box means no difference. M1, M2 and M3 represent MF at stages 5, 6 and 10, respectively, while H1, H2 and H3 represent bisexual flowers at stages 5, 6 and 10, respectively. The meaning of the ‘significant p-value’ was a significant difference between floral stages. Figure S3. Numbers of differentially expressed genes. (A) TERM1 – trend all by gene number, trend all by P-value. In profile 3: 2364 gene (2.6e-19 P-value) had stable expression in floral stages 5 and 6, but decreased in expression in stage 10; in profile 4, 1664 genes (1.1e-11 P-value) had stable expression in floral stages 5 and 6, but increased in expression in stage 10; in profile 5, 2320 genes (3.7e-17 P-value) increased in expression in floral stages 5 and 6, but decreased in stage 10; in profile 6, 2170 genes (6.2e-06 P-value) had an increased expression in floral stages 5 and 6, but had stable expression in stage 10; in profile 7: 1471 genes (7.7e-18 P-value) had an increased expression in flora [file 12870_2019_2081_MOESM4_ESM.zip › Fig. S4.jpg]
